# Interpretability, credibility, and usability of hospital-specific template matching versus regression-based hospital performance assessments; a multiple methods study

**DOI:** 10.1186/s12913-022-08124-w

**Published:** 2022-06-03

**Authors:** Brenda M. McGrath, Linda Takamine, Cainnear K. Hogan, Timothy P. Hofer, Amy K. Rosen, Jeremy B. Sussman, Wyndy L. Wiitala, Andrew M. Ryan, Hallie C. Prescott

**Affiliations:** 1 VA Center for Clinical Management Research, Ann Arbor, MI USA; 2 Department of Internal Medicine, University of Michigan, Ann Arbor, MI USA; 3 VA Center for Healthcare Organization and Implementation Research, VA Boston Healthcare System, Boston, MA USA; 4 Department of Surgery, Boston University School of Medicine, Boston, MA USA; 5 Department of Health Management and Policy, School of Public Health, University of Michigan, Ann Arbor, MI USA

**Keywords:** Quality of health care, Benchmarking, Risk adjustment, Hospital mortality

## Abstract

**Background:**

Hospital-specific template matching (HS-TM) is a newer method of hospital performance assessment.

**Objective:**

To assess the interpretability, credibility, and usability of HS-TM-based vs. regression-based performance assessments.

**Research design:**

We surveyed hospital leaders (January-May 2021) and completed follow-up semi-structured interviews. Surveys included four hypothetical performance assessment vignettes, with method (HS-TM, regression) and hospital mortality randomized.

**Subjects:**

Nationwide Veterans Affairs Chiefs of Staff, Medicine, and Hospital Medicine.

**Measures:**

Correct interpretation; self-rated confidence in interpretation; and self-rated trust in assessment (via survey). Concerns about credibility and main uses (via thematic analysis of interview transcripts).

**Results:**

In total, 84 participants completed 295 survey vignettes. Respondents correctly interpreted 81.8% HS-TM vs. 56.5% regression assessments, *p* < 0.001. Respondents “trusted the results” for 70.9% HS-TM vs. 58.2% regression assessments, *p* = 0.03. Nine concerns about credibility were identified: inadequate capture of case-mix and/or illness severity; inability to account for specialized programs (*e.g.*, transplant center); comparison to geographically disparate hospitals; equating mortality with quality; lack of criterion standards; low power; comparison to dissimilar hospitals; generation of rankings; and lack of transparency. Five concerns were equally relevant to both methods, one more pertinent to HS-TM, and three more pertinent to regression. Assessments were mainly used to trigger further quality evaluation (a “check oil light”) and motivate behavior change.

**Conclusions:**

HS-TM-based performance assessments were more interpretable and more credible to VA hospital leaders than regression-based assessments. However, leaders had a similar set of concerns related to credibility for both methods and felt both were best used as a screen for further evaluation.

**Supplementary Information:**

The online version contains supplementary material available at 10.1186/s12913-022-08124-w.

## Introduction

Benchmarking hospital performance is a cornerstone of hospital quality assessment [1]. However, differences in patient case-mix and illness severity must be accounted for in order to yield fair cross-hospital comparisons [1–3]. The most common approach to adjust for patient characteristics is to use regression models [3], but this approach has at least two key limitations. First, clinicians frequently question whether differences in patient populations have been sufficiently accounted for in regression models [4, 5], and this concern may limit the ability of regression-based performance assessments to drive positive change. Second, estimates from regression models are used to produce a standardized mortality ratio, which is a form of indirect standardization that compares an index hospital not directly to other hospitals, but to the other hospitals only if they were to admit hypothetical populations of patients similar to the index hospital [4]. Thus, no hospital is being judged against real patient care outcomes at other hospitals. As a result of these limitations, the National Academy of Medicine has recognized the need for greater transparency and interpretability of hospital benchmarking systems and called for dedicated research to improve the science of hospital performance assessment [6, 7].

Hospital-specific template matching (HS-TM) was proposed by Silber et al*.*^*4*^as a fairer and more transparent approach for assessing hospital performance. In this method, a representative sample of hospitalizations is selected from the hospital under evaluation, and the outcomes of the sampled hospitalizations are compared to outcomes of matched hospitalizations from a set of comparator hospitals with sufficiently similar patient case-mix to the hospital under evaluation [4, 8]. The performance assessment is thus customized for each hospital, providing a potentially fairer assessment than regression-based performance assessment [4, 8]. Furthermore, because the quality of matching can be readily reported, HS-TM provides greater transparency than regression [4]. In prior work, we have shown that HS-TM is feasible for hospital performance assessment in the Nationwide Veterans Affairs (VA) Healthcare system [8]. Despite the case-mix variation [9], VA hospitals could each be matched to enough comparator hospitals to support performance assessment across the entire system [8].

The statistical advantages and disadvantages of these two approaches have been explored in prior studies [4, 8]. However, while HS-TM has theoretical benefits over regression-based performance assessment and is feasible in the VA healthcare system [4, 8], it is unclear whether HS-TM is more interpretable, more credible, or more usable to end-users than the traditional regression-based performance assessments. Thus, in this study, we assessed the interpretability, credibility, and usability of HS-TM-based versus regression-based performance assessments among VA hospital leaders. To do this, we generated hypothetical hospital performance assessments using real VA patient data [8], then used surveys and semi-structured interviews of VA hospital leaders to assess the utility of HS-TM-based versus regression-based performance assessment. Interpretability and credibility were assessed quantitatively by survey. Actionability and specific concerns about credibility were assessed via semi-structured interviews.

## Methods

### Setting

The VA healthcare system is a large U.S. national integrated healthcare system for Veterans with approximately 130 hospitals, ranging from small rural hospitals to tertiary referral centers. VA has been a leader in the development and implementation of hospital performance assessment [2, 10, 11]. It was among the first healthcare systems to have an electronic health record and to measure and report risk-adjusted mortality [2, 10, 11]. VA mortality models are updated annually [12], and risk-adjusted 30-day mortality is a key outcome metric included in quarterly hospital performance assessments [12].

### Study design

We used a multiple methods approach to assess the interpretability, credibility, and usability of HS-TM-based versus regression-based hospital performance assessments among end-users charged with maintaining and improving the quality of VA care. We first surveyed VA hospital leaders (Chiefs of Staff, Chiefs of Medicine, and Chiefs of Hospital Medicine) to assess their ability to correctly interpret hospital performance assessments of risk-adjusted 30-day mortality and to evaluate their confidence in interpretation and trust in the assessment. (While no single metric is sufficient to evaluate hospital quality, we selected 30-day mortality as the outcome of interest in this study because of its importance to performance assessment in the VA system as well as in other healthcare systems.)

Second, we completed semi-structured interviews with a subset of Chiefs of Medicine to further explore their concerns regarding credibility and the uses of HS-TM-based versus regression-based performance assessments. eTable 1 summarizes the target population, enrollment, research tools, sample size, and analysis methods for the survey and the interviews. The study was approved by the Ann Arbor VA Institutional Review Board with a waiver of written documentation of informed consent for the survey portion. All methods were performed in accordance with relevant guidelines and regulations.

### Randomized survey

Chiefs of Staff, Chiefs of Medicine, and Chiefs of Hospital Medicine at approximately 130 nationwide VA hospitals were invited via group emails to complete an anonymous Qualtrics survey (Qualtrics, Provo, UT) from January through May 2021. Invitation emails were sent by VA leaders (*e.g.*, VA Ann Arbor Chief of Staff) to promote participation, with reminder and final invitation emails sent by study staff. No compensation was provided for survey completion since we anticipated surveys would be completed during respondents’ VA tour of duty.

The full survey is provided in Additional Appendix 1; key aspects of the survey are presented in Table 1. The survey vignettes were developed using 2017 VA hospitalization data [8]. The survey language was adapted from a prior survey assessing the presentation of quantitative information [13] and refined iteratively, incorporating feedback from 5 study co-investigators, each of whom participated in a 1-h cognitive interview. The survey was then piloted by 7 MD and 1 PhD-trained colleagues to determine the median time for completion (11 min) before deploying to hospital leaders.

**Table 1:**
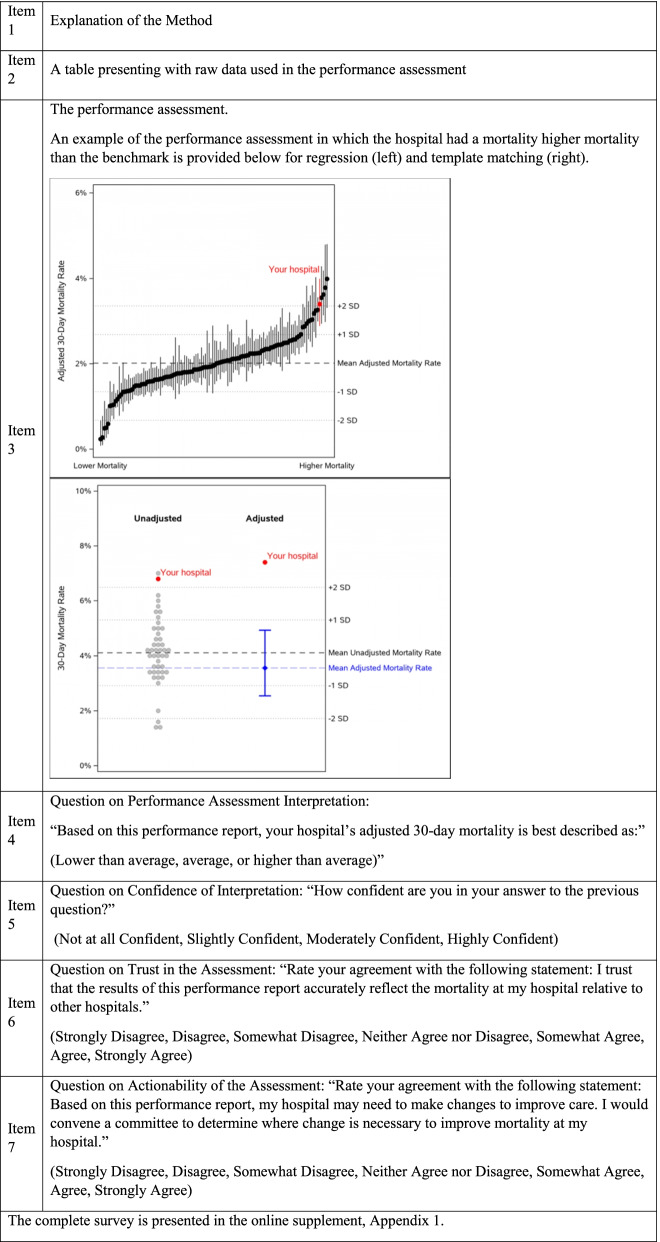
Six items included in each survey vignette

Each survey included four hypothetical performance assessments (for four hypothetical hospitals)—two using HS-TM and two using regression. Each survey included hypothetical hospitals across a range of 30-day mortality (one above-average, one high-average, one average, and one below-average risk-adjusted mortality). The order of performance methods (HS-TM versus regression) and mortality category (above-average, high-average, average, and below-average) were randomized. For each vignette, participants received a description of the performance assessment method, a table showing the characteristics of hospitalizations included in the performance assessment, and a figure displaying outcomes of their hospital relative to their comparator hospitals. Participants were asked to assess the hospital’s performance relative to their comparators (above-average, average (including high-average), and below average), then rate their confidence in interpretation and trust in the performance assessment on a Likert scale. At the end of the survey, participants were asked about their overall impressions of HS-TM versus regression-based performance assessment methods.

Survey results are presented using standard descriptive statistics and Chi-square tests to compare results of HS-TM vs regression-based vignettes. Secondly, a series of logistic regression models were fit to measure the association between the performance assessment approach (HS-TM vs regression) and correct interpretation. In the serial models, we additionally adjusted for the mortality category, the respondent’s self-rated statistical knowledge, and the respondent’s confidence in their response. The models included a random intercept for the respondent to control for the repeated measures.

### Semi-structured interviews

At the end of the survey, Chiefs of Medicine were asked to provide their contact information if amendable to participating in a confidential follow-up semi-structured interview. We invited only Chiefs of Medicine so that we would have just one interview participant per hospital. After completing written informed consent, Chiefs who expressed interest were invited for a 60-min semi-structured interview via video conference. The full interview guide is provided in Additional Appendix 2. During the interview, the participants were asked about two vignettes from their survey (one of each method), using an interview guide to elicit perceptions of credibility and usability. Additionally, we asked about interpretability, suggested improvements, and general impressions about performance evaluation. The interview guide was piloted with two physician colleagues and refined to improve clarity prior to use in the study.

Nine Chiefs of Medicine were interviewed via video conference. Interviews were audio-recorded, professionally transcribed, and redacted of identifying information. The sample size was guided by the criteria of “information power” [14]. We required fewer participants because the goal of the interviews was narrow; the participants were highly selected (limited to key leaders directly involved in evaluating hospital quality) [15, 16]; the feedback was anticipated to relate to known methodological limitations [3, 5–7]; and the interviews had high quality dialogue since they were conducted by an experienced, PhD-trained qualitative analyst (LT) with at least one quantitative expert (BMM and/or HCP) present to answer technical questions and probe responses as needed.

Interview transcripts were analyzed by LT, BMM, and HCP using content analysis [17]. We used preliminary codes (interpretability, credibility, usability, suggested improvements) based on the interview guide and allowed additional subcodes to emerge from the data. Transcripts were coded independently, then reconciled through discussion. Data were manually entered into separate code reports, which were reviewed and discussed as a team to finalize subcodes, summarize the key findings, and identify representative quotes.

## Results

Eighty-four VA hospital leaders completed at least one survey vignette (a response rate of approximately 21.5%), including 70 (83.3%) who completed all four vignettes and provided demographic data. Respondents included 17 (20.2%) Chiefs of Staff, 31 (36.9%) Chiefs of Medicine, and 36 (42.9%) Chiefs of Hospital Medicine. Descriptive characteristics of the respondents are presented in eTable 2. Respondents were 65.7% male. 52.9% were in their current role for 0–4 years, while 20.0% had been in their current role for $\ge$ 10 years. Length of time practicing medicine varied: 5.8% (0–9 years), 26.1% (10–19 years), 31.9% (20–29 years), and 36.2% (30 years or more). The majority (77.1%) rated their statistical knowledge as “Good” or “Fair”.

Respondents completed 148 vignettes using HS-TM, in which the hypothetical hospital under evaluation had below-average mortality (37, 25%), average mortality (39, 26.4%), high-average mortality (36, 24.3%), and above-average mortality (36, 24.3%). Respondents completed 147 vignettes using regression, in which the hypothetical hospital under evaluation had below-average mortality (37, 25.2%), average mortality (38, 25.9%), high-average mortality (39, 26.5%), and below-average mortality (33, 22.4%).

### Interpretability

**Fig. 1:**
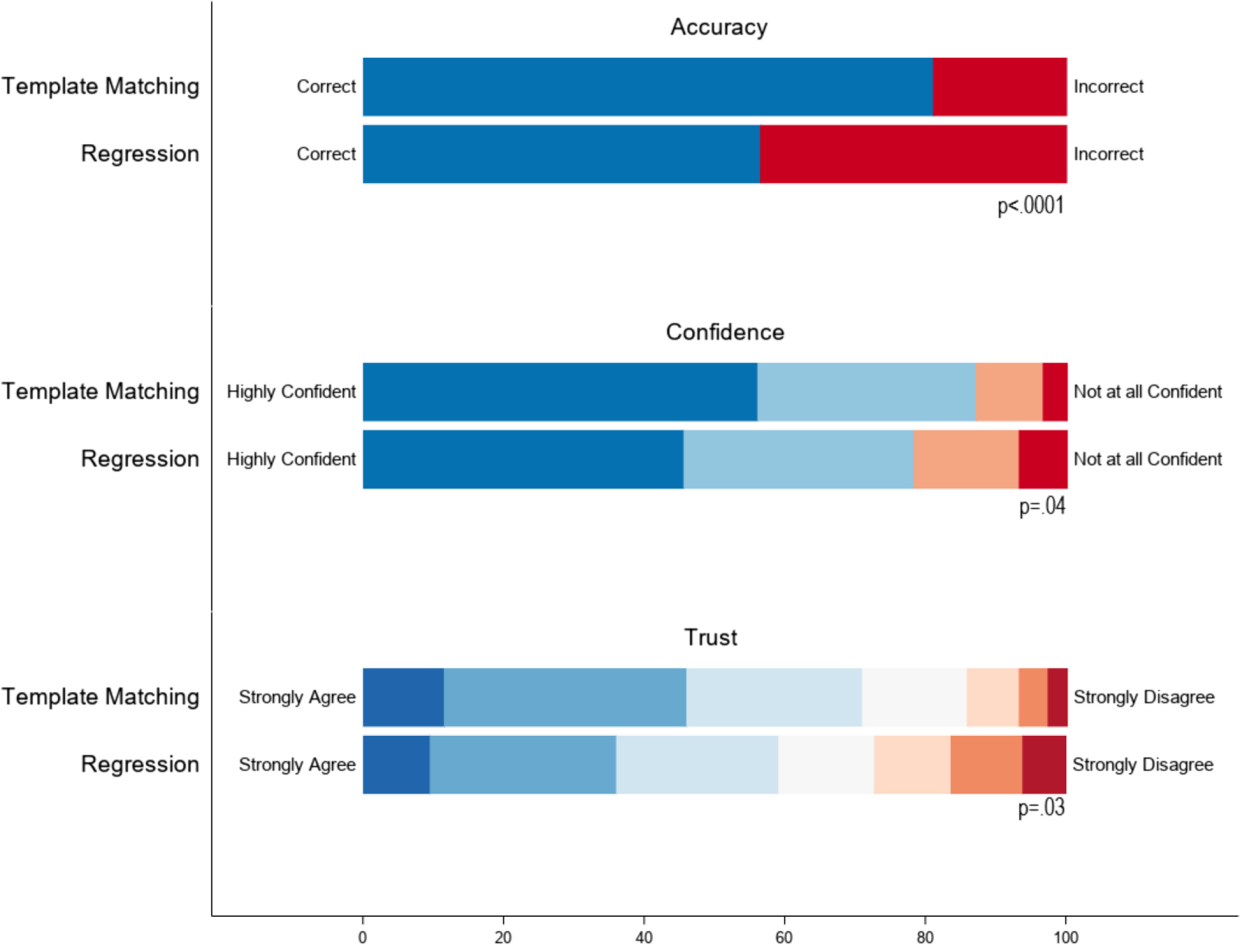
Accuracy, Confidence and Trust in the HS-TM-based vs Regression-Based Performance Assessments. Accuracy indicates whether the participant correctly classified the hospital as lower than average, average, or higher than average mortality. Confidence indicates how confident they were in their rating: Highly Confident, Moderately Confident, Slightly Confident, or Not at all Confident. Confidence is then dichotomized into Not Confident (Not at all Confident, Slightly Confident) or Confident (Moderately Confident, Highly Confident) and the p-value is the significance level of the difference in the percent Confident for HS-TM versus regression. Trust indicates their level of agreement with the following statement: I trust that the results of this performance report accurately reflect the mortality at my hospital relative to other hospitals. (Strongly Agree, Agree, Somewhat Agree, Neither Agree nor Disagree, Somewhat Disagree, Disagree, Strongly Disagree). The p-value indicates the significance level of the difference in the percent that trust the rating (Strongly Agree, Agree, or Somewhat Agree) using HS-TM versus regression

Respondents interpreted 81.8% of HS-TM vignettes vs. 56.5% of regression vignettes correctly, *p* < 0.001 (Fig. 1). Survey respondents determined the hospital’s performance correctly more often when the hospital’s mortality was above or below average (compared to being no different from average). For example, among HS-TM vignettes, respondents correctly interpreted 97.3% (36/37) of below-average mortality and 94.4% (34/36) of above-average mortality vignettes, compared to 74.4% (29/39) of average and 58.3% (21/36) of high-average HS-TM mortality vignettes (eTable 3, eFigure 1). For regression vignettes, respondents correctly interpreted 89.2% (33/37) of below-average mortality and 87.9% (29/33) of above-average mortality vignettes, compared to only 31.6% (12/38) of average morality and 23.1% (9/39) of high-average mortality vignettes (eTable 3, eFigure 1). After adjusting for hospital mortality, the association of HS-TM with correct interpretation was even stronger (Table 2) and persisted after additionally adjusting for the respondent’s self-rated statistical knowledge and confidence in their interpretation (Table 2). Neither self-rated statistical knowledge nor confidence were associated with correct interpretation (Table 2). Overall, these analyses show that HS-TM-based performance assessments were more interpretable to the survey respondents than the regression-based assessments.

**Table 2:** Serial logistic regression models assessing the association between approach (HS-TM vs regression) and correct interpretation of performance assessment vignettes

	Odds Ratio	95% CI	p	Mean Percent Correct	95% CI
**Model 1:** no covariates					
Approach					
Regression	ref			0.56	(0.47, 0.65)
HS-TM	3.62	(2.08, 6.28)	< .0001	0.82	(0.75, 0.88)
**Model 2:** scenario as a covariate					
Approach					
Regression	ref			0.63	(0.51, 0.74)
HS-TM	6.26	(3.10, 12.64)	< .0001	0.91	(0.85, 0.95)
Scenario^‡^					
Below-Average Mortality	17.70	(5.70, 54.98)	< .0001	0.95	(0.88, 0.98)
Average Mortality	ref			0.54	(0.40, 0.67)
High-Average Mortality	0.52	(0.24, 1.13)	0.10	0.38	(0.25, 0.53)
Above-Average Mortality	19.28	(5.75, 64.72)	0.91	0.96	(0.88, 0.99)
**Model 3:** scenario, self-rated statistical knowledge, and confidence as covariates					
Approach					
Regression	ref			0.59	(0.44, 0.72)
HS-TM	6.33	(3.10, 12.92)	< .0001	0.90	(0.81, 0.95)
Scenario					
Below-Average Mortality	19.41	(6.08, 61.91)	< .0001	0.95	(0.86, 0.98)
Average Mortality	ref			0.48	(0.33, 0.64)
High-Average Mortality	0.52	(0.24, 1.13)	< .0001	0.33	(0.20, 0.49)
Above-Average Mortality	21.06	(6.12, 72.52)	0.10	0.95	(0.86, 0.98)
Self-Reported Statistical Knowledge					
Poor	ref			0.78	(0.65, 0.87)
Good	1.05	(0.46, 2.42)	0.90	0.79	(0.63, 0.89)
Confidence in Assessment					
Not Confident	ref			0.72	(0.51, 0.86)
Confident	1.99	(0.76, 5.21)	0.16	0.83	(0.75, 0.90)

*CI* Confidence Interval

Across all 3 models the HS-TM approach was consistently associated with increased odds of correctly interpreting the performance assessment vignette

### Credibility

Survey respondents reported that they “trust that the results of the performance report accurately reflected the mortality at [their] hospital relative to other hospitals” in 70.9% of HS-TM vignettes versus 59.2% of regression vignettes, *p* = 0.03 for the difference (Fig. 1). Results stratified by mortality category are shown in eTable 3 and eFigure 2.

While survey respondents trusted most performance assessments (70.9% of HS-TM and 59.2% of regression vignettes), the interview participants voiced many concerns about the credibility of performance assessments—most which were pertinent to both HS-TM and regression. The concerns, presented in Table 3, related to the following domains: (1) the inability to fully or correctly capture case-mix and illness severity from the electronic health record; (2) the inability to account for special hospital programs or referral centers (*e.g.*, an organ transplant center where many patients with end-stage disease may be evaluated but not ultimately eligible for transplantation); (3) the comparison to hospitals elsewhere in the country, as opposed to VA or non-VA hospitals in the same geographic region; (4) the use of mortality as a measure of quality; (5) lack of a criterion or reference standard for acceptable or good performance; (6) small sample sizes and/or low event rates, such that assessments are under-powered and unstable; (7) the comparison to dissimilar hospitals (*e.g.*, comparison of an urban referral hospital to a smaller rural hospital); (8) the generation of hospital rankings, particularly when hospitals are tightly clustered such that differences in rank do not necessarily reflect differences in outcomes; (9) the lack of transparency of performance assessments. Concerns 1–5 were equally relevant to both approaches. Concerns about small sample size were more pertinent to HS-TM, while concerns about ranking [8], lack of transparency, and comparison of dissimilar hospitals were more pertinent to regression. A fuller summary of interview responses related to fairness and credibility is presented in Additional Appendix 3.

**Table 3:** Concerns about the credibility of hospital performance assessments identified through thematic analysis of interview transcripts

Concern		Representative Comments
Concerns inherent to both regression and HS-TM		
1	**Case-mix and illness severity.** Participants felt that incomplete or inaccurate clinical documentation, laboratory testing, and diagnostic coding would under-estimate case-mix or illness severity, which could impact performance assessments	“I could look at a hospital that is two standard deviations above and say they’re just not doing a good job of coding.” “it’s nice to verify elements periodically…if our folks aren’t documenting with accuracy. A few years back, we had 75% of our pneumonias being coded as pneumonias not otherwise specified, so the bulk of that was our providers not being appropriately detailed consistently in their documentation, so we addressed that.” “we may find that we’re not doing a good job documenting our comorbidities, right? The model is dependent on comorbidities, and so, if we’re not doing a good job documenting those, maybe that’s a factor that’s driving our performance as opposed to saying oh there’s something wrong with the quality of our care.”
2	**Special hospital programs or referral centers**. Participants felt that performance assessments cannot account for the existence of special hospital programs or referral centers that are associated with treating patients with advanced disease	“as a transplant hospital, you cannot account for a very select population of patients that are five times the standard deviation beyond the mean when they come into your hospital essentially dying and they’re there to try to get a lifesaving action, so I do need to consider some of the nuances of our hospital.” “we are a major cancer center, so we have a lot of patients referred to us for cancer care throughout the state. Literally we are physically attached to the only accredited cancer center in the state, so …you certainly have to take those nuances into consideration.” “one hospital’s cardiovascular admissions might be quite different from hospital to hospital depending on the sort of services that are being offered. We’re a tertiary or eve quaternary facility here, and so, even if you look at some of the cases we do of coronary interventions or [other advanced cardiac procedure], you know the surgery that we do here that isn’t offered at some of the other facilities, the admission diagnostic group might be the same, but the complexity of the patients might be different.”
Concerns pertinent to both regression and HS-TM, but inherent to neither method		
3	**Geographically disparate comparators**. Participants felt comparisons to local hospitals had more face validity than comparisons to geographically disparate hospitals	“we have that problem locally already where we get benchmarked against other VA hospitals, but not necessarily against our local cohort … So, for instance, my hospital isn’t necessarily benchmarked against the hospital down the street from me, they’re benchmarked against other VA hospitals with similar cohorts of things that you’re showing me here, but I lose that sort of local flavor which might actually make a difference…I still am convinced that there is a local flair to certain data that may not necessarily get accounted for.” “a patient with diabetes in [city a] may not be the equivalent of a patient with diabetes and [city b], because of other confounding factors like being in a rural area or being poor or—who knows—I could come up with probably dozens of potential confounders, so how well the model accounts for those things is important”
4	**Mortality as a quality measure**. Participants were reticent to equate low mortality with quality	“Well, I wouldn’t say that [the performance report’s] necessarily effective at describing the quality of care. It’s just telling me what my mortality rate is compared to the mean. So, I think it might be a leap to conclude that the problem may be quality of care” …
5	**Lack of criterion or reference standard.** Participants preferred criterion standards over norm references	“what we’re really doing is we’re seeing how we’re compared to everybody else, which means there’s going to be—I don’t want to say it—but there’s going to be a winner and there’s going to be a loser, right. There’s going to be a number one and there’s going to be somebody who’s at the end. The way I likened stuff like this is, if you took the top 10 violinists on the planet, the top 10 people who you felt were the greatest violinists, they were just amazing, and you applied SAIL to them, one of them would be a one-star violinist right?” “I prefer to say: here is the standard, and let’s all measure ourselves against what we think is the standard.” “if you keep doing norm referencing…someone’s always going to end up on top, and on the bottom, and they are going to feel terrible or really great about it, and it’s not necessarily true… you know our standard wait times are so much less than places in the Community that it’s embarrassing for the Community…but my wait time may be the worst in the country for VA…so this starts to not make sense at some point.”
Concerns more pertinent to HS-TM than regression		
6	**Small sample sizes and/or low event rates.** Participants primarily worried that low sample size and event rates would yield unstable assessments	“Well, 500 hospitalizations. This you know isn’t all that many in my mind…for each of these groupings, you end up with a relatively smaller number of patients to look at, and you know if you’re looking at a mortality rate of let’s just say it’s 3% and 500 you know that’s 15 people that died, right?” “So, it’s just that in a year’s period of time, the number of deaths we will have is not that great, and so even though I’m trying to adjust for all these factors, if in the next six month period for any random reasons I have three or four more deaths, I could suddenly be very close to the you know the 30 day mean with just a small with a relatively small number”
Concerns more pertinent to regression that HS-TM		
7	**Dissimilar comparator hospitals**. Participants felt hospitals should be compared to “true peers” rather than the system at large	“[hospital-specific template matching is] getting closer to this idea of comparing yourself to other hospitals that are that are of the same ilk” “It just seems to make more sense to me to compare like facilities that are similar more so than trying to adjust facilities that are dissimilar and make them similar.”
8	**Generation of hospital rankings.** Participants felt that rankings rarely reflect meaningful differences in outcomes	“you don’t know sometimes how tightly things are grouped. We had one quarter, where we move from fifth quintile to second in an acute MI measure… we looked and everybody was packed in there and it was four thousandths of a point difference that moved us about 80 ranking points…” “when the VA chooses to rank things from the top performing hospital to the lowest performing hospital, being a low performer in that type of ranking doesn't necessarily mean that you're performing badly” “I kind of look at the tails more than if I’m clustering the middle, I really don't know if I can distinguish whether facilities within that interquartile range of 25/75 are materially different from one another.”
9	**Lack of transparency.** Participants felt performance assessments sometime lack sufficient detail to be helpful	“there is certainly a habit of data coming down from above telling us we have a problem to fix it without us really having the ability to see what the data really is that goes into that assessment. We’re in the midst of that right now, where I’m told, something that I don’t agree with because, you know, as the person who looks at every admission every day I don’t see this, and so I don’t understand how our metric is so bad when I’m actually looking every day at what’s walking in the door and either I’m not measuring it correctly or I don’t understand what goes into the metric. And I need that information so that I can better understand whether there truly is a problem and how we would go about addressing it.”

### Usability

Survey respondent agreed with the statement “Based on this performance report… I would convene a committee to determine where change is necessary to improve mortality at my hospital”, for 88.9% of HS-TM vignettes with above-average mortality, compared to 78.7% regression vignettes with above-average mortality (*p* = 0.25 for difference)—suggesting similar actionability of HS-TM vs regression-based assessments.

Survey participants described two primary uses of performance assessments: (1) to trigger a deeper dive and (2) to motivate behavior change (Table 4, Additional Appendix 4). Interview participants reported that they would use both HS-TM-based and regression-based performance reports similarly, but several expressed that HS-TM may be more helpful for identifying a true problem, while the ranking generated by regression-based performance assessments may be more helpful for motivating behavior change (eTable 4).

**Table 4:** Usability of Performance Assessments

	**Use**	**Representative Comments**
1	**Trigger a deeper dive** to understand the data	“if people were dying at a higher rate at my hospital, I wouldn't say that means that we're providing poor quality care. I think what it is, is it's a trigger to say, why are people dying. It's a trigger for a deeper dive.” “it's a little bit like ‘check oil’. There's a lot of reasons why that light may come on and so you need to get under the hood to understand why that that check oil light is coming on. So, I would never devote a huge amount of resources without having a good understanding of why we might be an outlier.” “[these data are] a flag or an indicator for something that that we might need to respond to” “in and of itself, the data doesn't say you're good, bad, or indifferent” “this would be enough for me to start trying to understand why does this exist” … are their service lines, care processes that that we need to be focusing improvement efforts on to bring these numbers down
2	**Galvanize stakeholders** and motivate behavior change	“it's possible that I might use this to impress upon certain stakeholders that this is indeed something that we need to devote some energy to” “it's been very well shown that if you want to motivate physician performance just show them where they stand as compared to their peers and they don't like to be [at the bottom]. You know, it's like lake Wobegon, 90% want to be in the top 10% of their class.” “what changes physician behavior in my experience, more than anything is a comparison to your peers in your hospital. I’ve been struck by how that's been true in different organizations, because you can look at a study and say, well, my patients are sicker or older, or they live further away, so I can't discharge them as early, but when you look at how your peers, are doing with the same patients in the same organization you start having to own the differences more, so some providers-specific data on outcomes could be an asset in prompting change”

A common sentiment among interview participants was that “in and of itself, the data doesn't say you're good, bad, or indifferent”. Rather, above-average mortality was consistently viewed as a trigger for further evaluation, described my participants as “a flag or an indicator for something that that we might need to respond to”, a “trigger for a deeper dive”, a “red flag”, or a “check oil light”. Most interview participants felt the deeper dive should occur to confirm and understand the potential issues raised in performance assessment before sending it to clinical staff. As a first step, interview participants would explore whether deaths were occurring on a specific service (*e.g.*, medical vs. surgical) or subgroups of patients (*e.g.*, ICU vs non-ICU), or even complete chart reviews of all deaths. They would consider unique circumstances related to their patient population or any specific care-related practices. In short, they would evaluate who died, why they died, and how they died to assess whether greater-than-average mortality was a one-time occurrence, a reflection of natural variation over time, or a marker of a broader problem. All interview participants felt it was inappropriate to use performance assessments for punishment or reward.

Besides serving as a trigger for a deeper dive, multiple interview participants reported that greater-than-average mortality can serve as strong motivation to improve processes and help one “get on it with a sense of urgency” and “impress upon certain stakeholders that this is indeed something that we need to devote some energy to… particularly if we find that there is a certain service line that seems to be over-represented in our mortality”. Finally, participants also noted that assessments indicating a mortality at or below the mean should not trigger complacency. Rather, hospitals should always look for opportunities to improve, although there is less urgency to do so when performance assessments suggest average or below average mortality.

### Suggestions for improvement

Suggested improvements are presented in Additional Appendix 5. The most common suggestions were to: (1) use criterion standards rather than norm-reference (particularly since non-VA hospitals are not used to define the norm-reference) and (2) limit comparisons to similar hospitals, as defined by facility characteristics or geographic location.

### Overall utility

When asked which method would be “more helpful for understanding mortality at your hospital relative to other hospitals”, most (72.5%, 50/69) survey respondents preferred HS-TM. Likewise, when asked which method would be “more helpful for driving change to improve care at your hospital”, most preferred HS-TM (78.3%, 54/69). Regarding distinctive features of these methods, 88.4% responded it was more important to be compared to hospitals treating similar patients (as in HS-TM) than to have all hospitalizations included in the performance assessment (as in regression). During semi-structured interviews, several participants expressed greater trust in HS-TM assessments, but participants nonetheless felt that—regardless of the method—they would primarily use performance assessments as a screen for doing a deeper dive. A summary of comments comparing the utility of HS-TM to regression is presented in eTable 4.

## Discussion

Hospital performance assessment is a key tool for monitoring the quality of hospital care and incentivizing performance improvement. However, while the breadth and complexity performance assessment has grown over the past few decades, there has been little assessment of the interpretability, credibility, or usability of performance assessments among the end-users charged with maintaining and improving the quality of hospital care [6]. Indeed, a National Academy of Medicine expert panel called for improving the robustness of performance assessment systems, including settings thresholds for interpretability such that assessments are understandable and usable by those with limited statistical knowledge and time [6].

We found that hospital performance assessments developed using hospital-specific template matching were more interpretable and more credible to VA hospital leaders than performance assessments developed using regression. The greater interpretability of hospital-specific template matching was robust to sensitivity analyses. Across a series of models including adjustment for additional factors including the mortality category of the hospital under evaluation, the respondent’s self-rated statistical knowledge, and the respondent’s self-rated confidence in their interpretation, HS-TM remained associated with increased likelihood of correct interpretation.

A second finding of this study was that hospital performance assessment served two key purposes in the perspective of VA hospital leaders: a trigger for further quality investigation and a tool for motivating behavior change. Among interview participants, HS-TM was generally considered to be a more reliable trigger, while hospital rankings generated by regression were considered more helpful for motivating behavior change. As a result of these differing strengths, HS-TM could be considered as a supplement or adjunctive method rather than a replacement for standard regression-based assessments. Importantly, the Chiefs of Medicine identified many potential threats to the credibility of both methods, and universally felt that further evaluation of the accuracy of performance assessments was needed before passing along the findings to front-line clinical staff.

This study extends the findings of prior studies of HS-TM. We previously showed that HS-TM was potentially feasible for use in the diverse VA healthcare system [8]. Each hospital could be matched to a sufficient number of comparison hospitals (median 38 hospitals) to detect standardized mortality ratios greater than 2.0 [8]. Here, we show that assessments generated via HS-TM are more interpretable and credible to VA hospital leaders. Our study also builds on limited prior work assessing clinician end-user’s ability to correctly interpret performance assessments. In a prior study examining clinicians’ interpretation of central line-associated bloodstream infection (CLABSI) quality data, clinicians answered questions testing increasingly difficult domains of interpretability: basic numeracy, risk-adjustment numeracy, and finally risk-adjustment interpretation [18]. Clinicians answered 82% of basic numeracy questions correctly, versus 70% of risk-adjustment numeracy and only 43% of risk-adjustment interpretation questions, underscoring the limited interpretability of risk-adjusted performance assessment among end-users [18]. Also concerning, respondents who accurately interpreted the data were more likely to view it as unreliable [19]. Our finding that HS-TM (which uses matching rather than regression adjustment to account for case-mix differences) was more interpretable than regression is consistent with this prior study showing limited interpretability of risk-adjusted data. However, reassuringly, HS-TM was not only associated with greater interpretability, but also with greater credibility.

Finally, our study is consistent with the broader literature on quantitative data interpretation. End-users have better comprehension and make better decisions when information is presented in a way that is easier to process and understand [20]. And, while the simplicity of data presentation is particularly important for individuals with low numeracy, even high numeracy individuals perform better when presented simpler information. Indeed, our study showed no association between self-rated statistical knowledge and correct interpretation of the performance assessment vignettes.

Our study should be interpreted in the context of several limitations. First, our survey response rate was approximately 21.5%, and it is possible that survey respondents may not generalize to VA leaders at large. However, our survey sample population was highly selected and relatively homogenous (limited to Chiefs of Staff, Chiefs of Medicine, and Chiefs of Hospital Medicine), which may mitigate the risk for bias due to the lower response rate. Second, we interviewed leaders within the VA healthcare system only, so it is unclear whether hospital leaders in other healthcare systems or countries would have similar reactions to HS-TM vs regression. However, the VA is a large and diverse system, with both small rural hospitals and tertiary referral centers [9]; interview participants represented a range of hospital types. One key benefit is the ability to personalize the assessment to diverse hospitals. A second key benefit is the improved interpretability. In a healthcare system or country where similar patient populations are treated across all hospitals, the benefits of a personalized assessment may be less important. However, such homogeneity is rare. Third, survey respondents were provided hypothetical vignettes, and it is possible that impressions of credibility may differ if HS-TM were used in practice. We decided to use hypothetical vignettes to randomize the hospital mortality category and differentiate the impact of the method vs mortality category on impressions of credibility, which would not have been possible using each respondent’s own hospital data. Fourth, we assessed only one quality outcome, mortality. Hospital quality is a complex and multi-faceted construct [21] which cannot be summarized by hospital mortality alone, or by any single metric. However, mortality is a key performance indicator, and the methods of HS-TM and regression can be applied to other outcomes such that the findings of improved interpretability and credibility are not necessarily specific to mortality only.

## Conclusion

In this multiple methods study of VA hospital leaders, HS-TM-based performance assessments were more interpretable and more credible than regression-based assessments. However, both types of assessments had several threats to credibility and would be used for similar purposes by hospital leaders. The differing interpretability and credibility across performance assessment methods underscores the importance of evaluating, understanding, and optimizing interpretability and credibility of performance assessments among end-users.

## Supplementary Information

**Additional file 1.**

## Data Availability

The datasets generated and/or analysed during the current study are not publicly available due the specifications of our IRB approval; however, annonomyzed data are available from the corresponding author on reasonable request.

## References

[1] Escobar GJ, Greene JD, Scheirer P, Gardner MN, Draper D, Kipnis P (2008). Risk-adjusting hospital inpatient mortality using automated inpatient, outpatient, and laboratory databases. Med Care.

[2] Render ML, Kim HM, Welsh DE (2003). Automated intensive care unit risk adjustment: results from a National Veterans Affairs study. Crit Care Med.

[3] Silber JH, Rosenbaum PR, Ross RN (2014). Template matching for auditing hospital cost and quality. Health Serv Res.

[4] Silber JH, Rosenbaum PR, Ross RN (2014). A hospital-specific template for benchmarking its cost and quality. Health Serv Res.

[5] Lezzoni LI (1997). The risks of risk adjustment. JAMA.

[6] Austin JM, McGlynn EA, Pronovost PJ (2016). Fostering transparency in outcomes, quality, safety, and costs. JAMA.

[7] Pronovost PJ, Austin JM, Cassel CK, et al. Fostering Transparency in Outcomes, Quality, Safety, and Costs: A Vital Direction for Health and Health Care | National Academy of Medicine. 2016; 10.1001/jama.2016.14039 27669332

[8] Vincent BM, Molling D, Escobar GJ (2021). Hospital-specific template matching for benchmarking performance in a diverse multihospital system. Med Care.

[9] Molling D, Vincent BM, Wiitala WL (2020). Developing a template matching algorithm for benchmarking hospital performance in a diverse, integrated healthcare system. Medicine (Baltimore)..

[10] Fihn SD, Francis J, Clancy C (2014). Insights from advanced analytics at the Veterans Health Administration. Health Aff (Millwood).

[11] Render ML, Deddens J, Freyberg R (2008). Veterans Affairs intensive care unit risk adjustment model: validation, updating, recalibration. Crit Care Med.

[12] Prescott HC, Kadel RP, Eyman JR, et al. Risk-Adjusting Mortality in the Nationwide Veterans Affairs Healthcare System. J Gen Intern Med. Jan 13 2022;doi:10.1007/s11606-021-07377-1 10.1007/s11606-021-07377-1 PMC9640507 35028862

[13] Hawley ST, Zikmund-Fisher B, Ubel P, Jancovic A, Lucas T, Fagerlin A (2008). The impact of the format of graphical presentation on health-related knowledge and treatment choices. Patient Educ Couns.

[14] Malterud K, Siersma VD, Guassora AD (2016). Sample size in qualitative interview studies: guided by information power. Qual Health Res.

[15] Marshall MN (1996). Sampling for qualitative research. Fam Pract.

[16] Hamilton AB, Finley EP (2019). Qualitative methods in implementation research: an introduction. Psychiatry Res.

[17] Hsieh HF, Shannon SE (2005). Three approaches to qualitative content analysis. Qual Health Res.

[18] Govindan S, Chopra V, Iwashyna TJ (2017). Do Clinicians Understand Quality Metric Data? An Evaluation in a Twitter-Derived Sample. J Hosp Med.

[19] Govindan S, Wallace B, Iwashyna TJ, Chopra V (2018). Do Experts Understand Performance Measures? A Mixed-Methods Study of Infection Preventionists. Infect Control Hosp Epidemiol.

[20] Peters E, Klein W, Kaufman A, Meilleur L, Dixon A (2013). More Is Not Always Better: Intuitions About Effective Public Policy Can Lead to Unintended Consequences. Soc Issues Policy Rev.

[21] Carini E, Gabutti I, Frisicale EM (2020). Assessing hospital performance indicators. what dimensions? evidence from an umbrella review. BMC Health Serv Res..

