# Supplementary material for: Interpretability, credibility, and usability of hospital-specific template matching versus regression-based hospital performance assessments; a multiple methods study
Source: BMC Health Serv Res. 2022 Jun 3;22:739. doi: 10.1186/s12913-022-08124-w (PMC9166576; doi:10.1186/s12913-022-08124-w)
Supplement: Supplementary file 1 — Additional file 1. [file 12913_2022_8124_MOESM1_ESM.docx]

**Supplement to:** Interpretability, Credibility, and Usability of Hospital-Specific Template Matching versus Regression-based Hospital Performance Assessment; A Multiple Methods Study

**Authors**: Brenda M. McGrath, MS; Linda Takamine, PhD; Cainnear K. Hogan, MSW; Timothy P. Hofer, MD, MSc; Amy K. Rosen, PhD; Michael Shwartz, PhD, MBA; Jeremy B. Sussman, MD, MS; Wyndy L. Wiitala, PhD; Andrew M. Ryan, PhD; and Hallie C. Prescott, MD, MSc

| **Online Supplement Table of Contents** |  |
| --- | --- |
| **eTable 1:** Summary of Research Design | Page 2 |
| **Appendix 1:** Qualtrics Survey Questions | Page 3 |
| **Appendix 2**: Interview Guide for Semi-Structured Interviews | Page 31 |
| **eTable 2**: Descriptive Characteristics of Survey Participants | Page 35 |
| **eTable 3**: Survey results for HS-TM-based vs regression-based performance assessment vignettes, stratified by mortality category | Page 36 |
| **eFigure 1**: Interpretabity of HS-TM-based vs regression-based performance assessment vignettes, stratified by mortality category | Page 37 |
| **eFigure 2:** Trust in HS-TM-based vs regression-based performance assessment vignettes, stratified by mortality category | Page 38 |
| **Appendix 3**: Summary of interview responses regarding fairness and credibility | Page 39 |
| **Appendix 4:** Summary of interview responses regarding usability | Page 41 |
| **Appendix 5:** Summary of interview responses regarding opportunities for improvement | Page 43 |
| **eTable 4:** Qualitative statements comparing the utility of HS-TM vs regression-based performance assessments, providing in response to the probe | Page 44 |

| **eTable 1**: Summary of Research Design | | |
| --- | --- | --- |
|  | **Phase 1: Survey** | **Phase 2: Semi-structured interviews** |
| **Target Population** invited to participate in the research | All Chiefs of Staff, Chiefs of Medicine, and Chiefs of Hospital Medicine in nationwide Veterans Affairs Healthcare System | All Chiefs of Medicine who completed the phase 1 survey and provided contact information in order to be contacted for a follow-up semi-structured interview. |
| **Number Enrolled** | Of an estimated 390 hospital leaders invited to participate, 84 completed the survey. | Of 31 Chiefs of Medicine who completed the survey, 10 indicated willingness to complete a follow-up semi-structured interview, of whom 9 completed the interview (1 never returned the informed consent document so could not be enrolled). |
| **Research Tools** | A randomized survey with 4 hypothetical hospital vignettes. The full survey is presented in **Appendix 1**. | Interviews were completed to follow-up on the survey and further explore concerns related to interpretability and credibility of the two performance assessment methods. The semi-structured interview guide is presented in **Appendix 2**. |
| **Power and sample size** | We estimated that with 109 respondents and 70% correct response to regression-based vignettes, we would have 80% power to detect a 14% absolute difference in interpretation. In the study, there were 84 respondents, 57% correct response to regression-based vignettes and a 25.3% difference in correct interpretation, which was statistically significant. | Given that this was a qualitative analysis, there were not formal power calculations. However, the sample size was guided by the criteria of “information power”.[^1^](#_ENREF_1) We required fewer participants because the goal of the interviews was narrow; the participants were highly selected (limited to key leaders directly involved in evaluating hospital quality);[^2^](#_ENREF_2)^,^[^3^](#_ENREF_3) the feedback was anticipated to relate to known methodological limitations;[^4-7^](#_ENREF_4) and the interviews had high quality dialogue since they were conducted by an experienced, PhD-trained qualitative analyst (LT) with at least one quantitative expert (BMM and/or HCP) present to answer technical questions and probe responses as needed. |
| **Method of Analysis** | We present survey results using standard descriptive statistics and compare results of HS-TM vs regression-based vignettes using Chi-square tests. We then fit a series of logistic regression models to measure the association between the performance assessment approach (HS-TM vs regression) and correct interpretation. In the serial models, we additionally adjusted for the mortality category, the respondent’s self-rated statistical knowledge, and the respondent’s confidence in their response. The models included a random intercept for the respondent to control for the repeated measures. | Interview transcripts were analyzed by LT, BMM, and HCP using content analysis.[^8^](#_ENREF_8) We used preliminary codes (interpretability, credibility, usability, suggested improvements) based on the interview guide and allowed additional subcodes to emerge from the data. Transcripts were coded independently, then reconciled through discussion. Data were manually entered into separate code reports, which were reviewed and discussed as a team to finalize subcodes, summarize the key findings, and identify representative quotes. |

|  | **Approach** | |
| --- | --- | --- |
| **Mortality Category** | HS-TM | Regression |
| Below average | Vignette 1a | Vignette 1b |
| Average | Vignette 2a | Vignette 2b |
| High average | Vignette 3a | Vignette 3b |
| Above average | Vignette 4a | Vignette 4b |

**Appendix 1:** Qualtrics Survey Questions

We created 8 total vignettes, as show in the table. Each survey contained 4 of the 8 total vignettes, two of which were HS-TM and two of which regression, with one vignette from each mortality category. The order of approach and mortality category were randomized for each survey using the Qualtrics randomization feature. For example, one survey could present vignettes 1a, 3a, 4b, 2b; another survey could present 4b, 1b, 3a, 3b. Below we present all 8 vignettes, as viewable to survey respondents in Qualtrics.


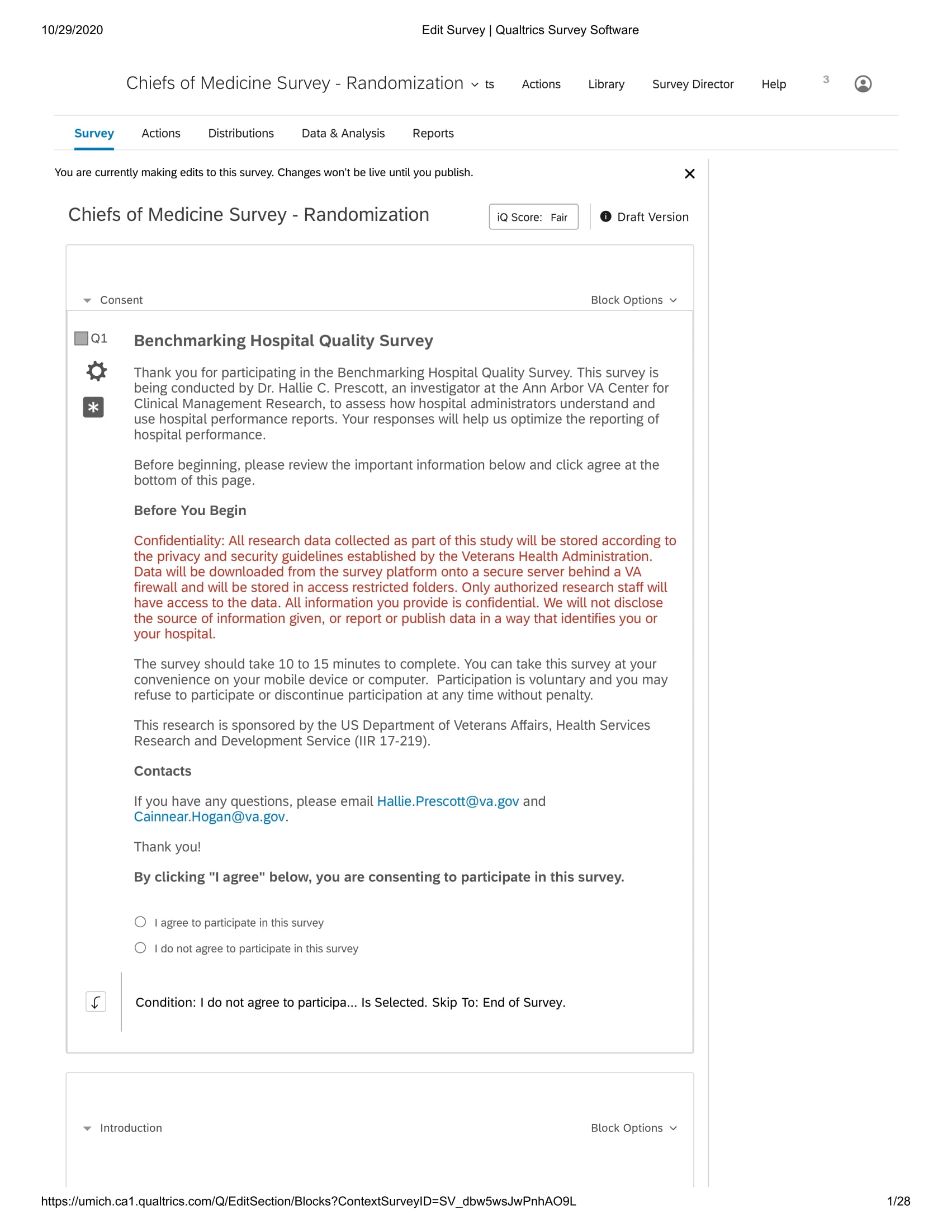


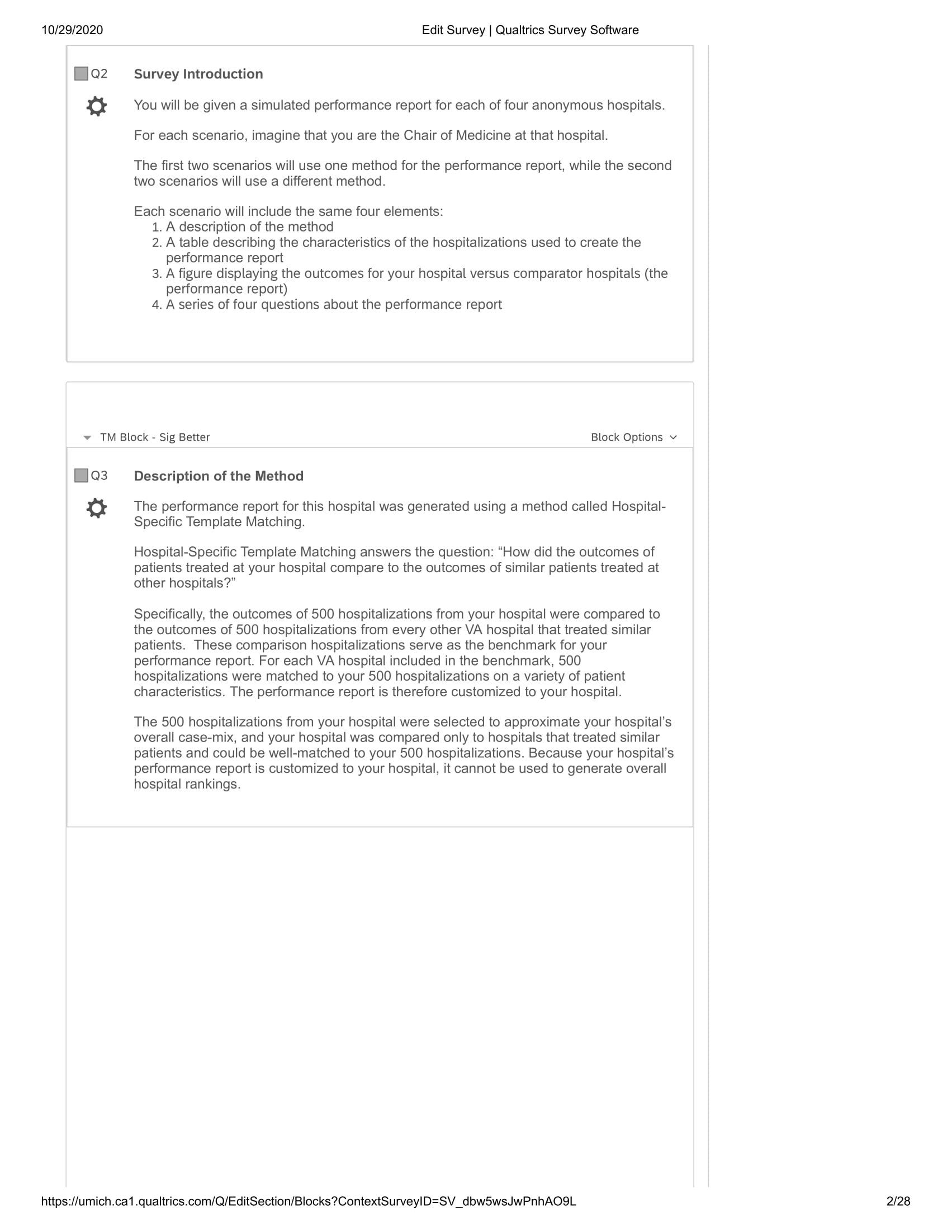


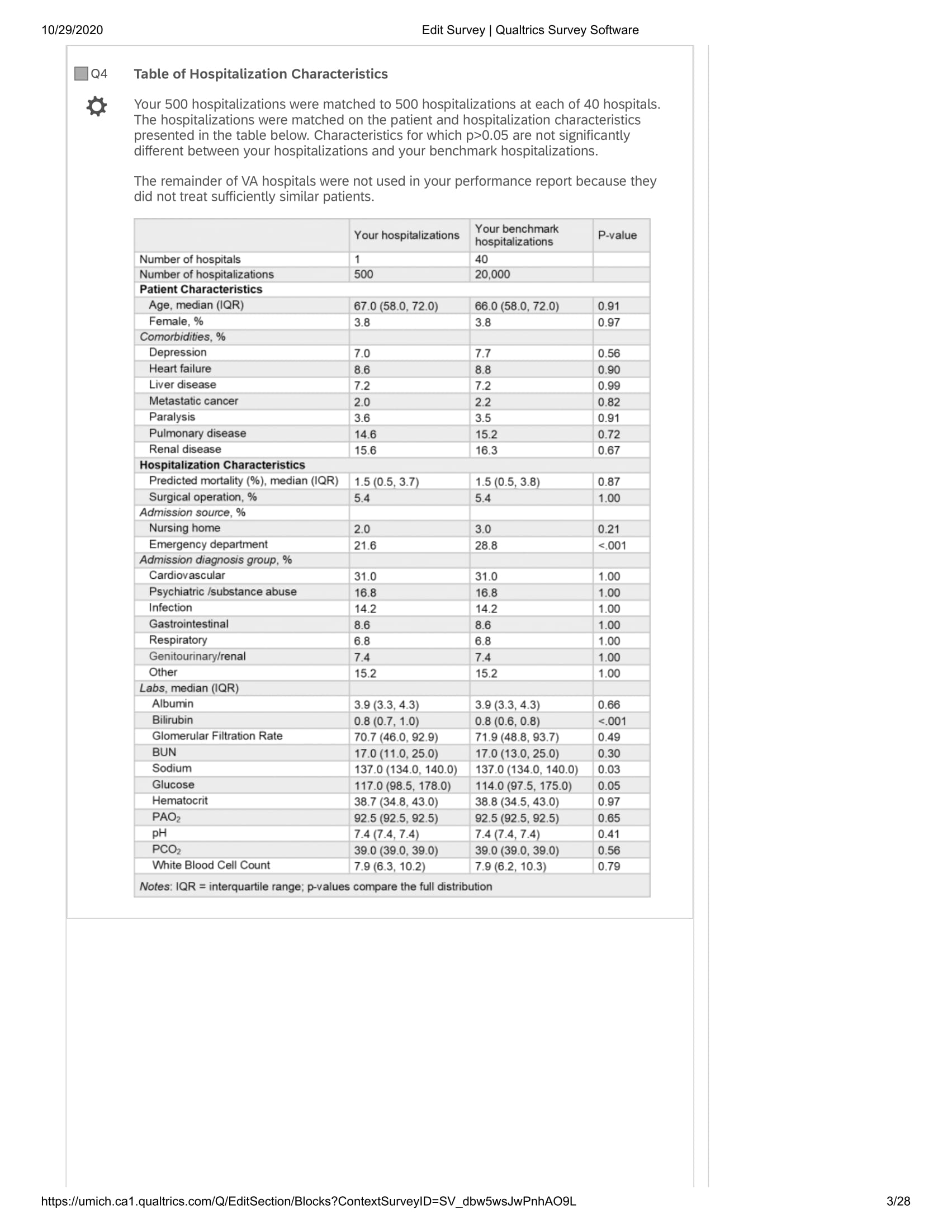


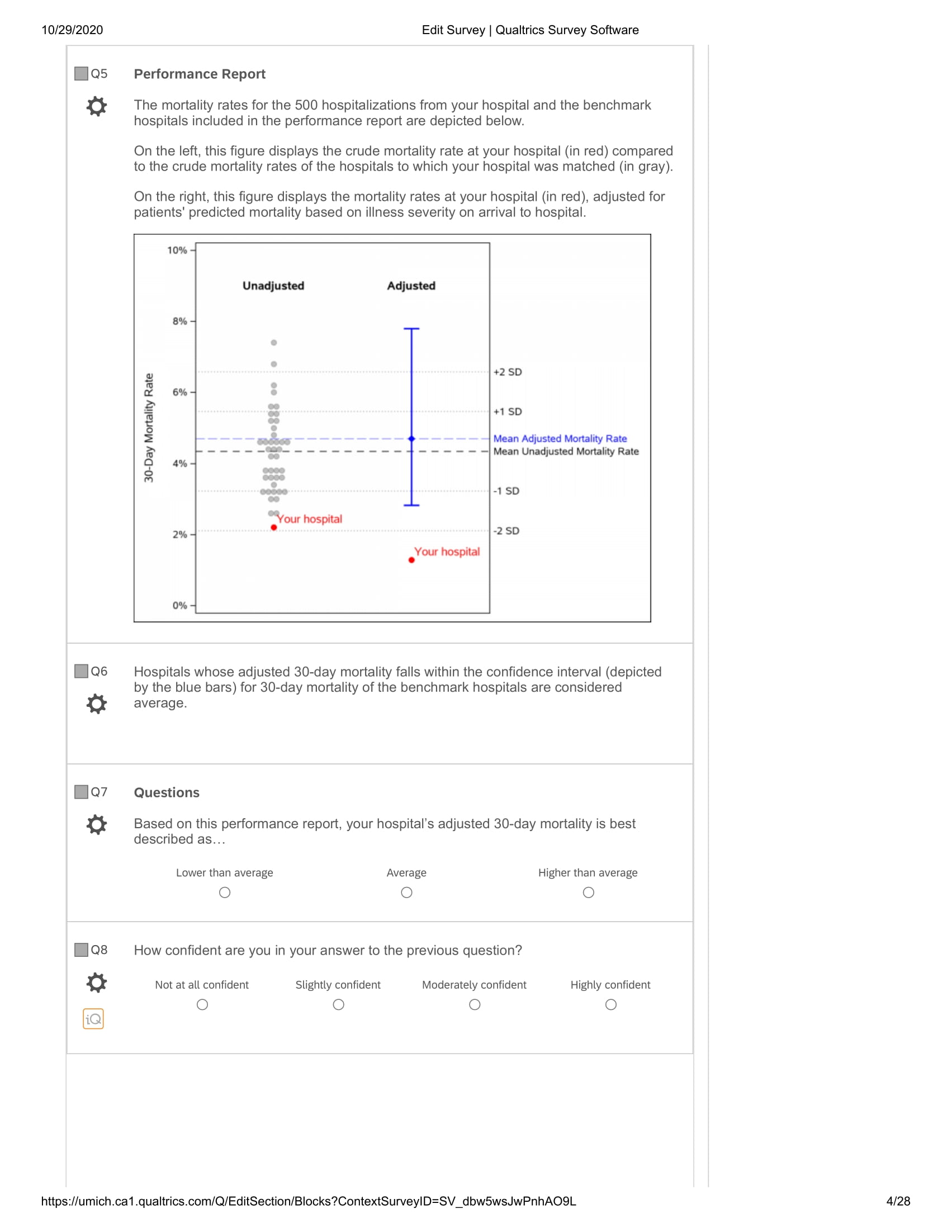


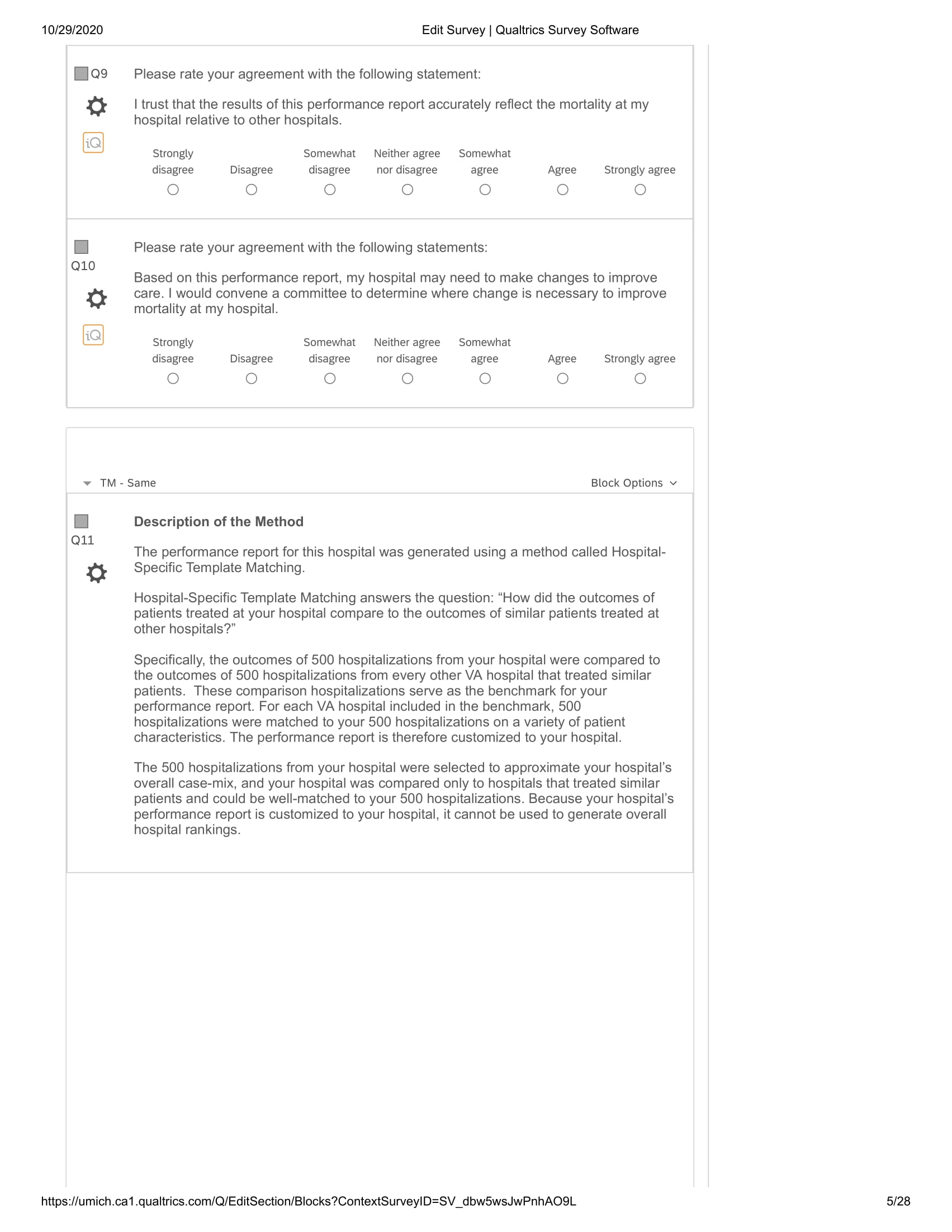


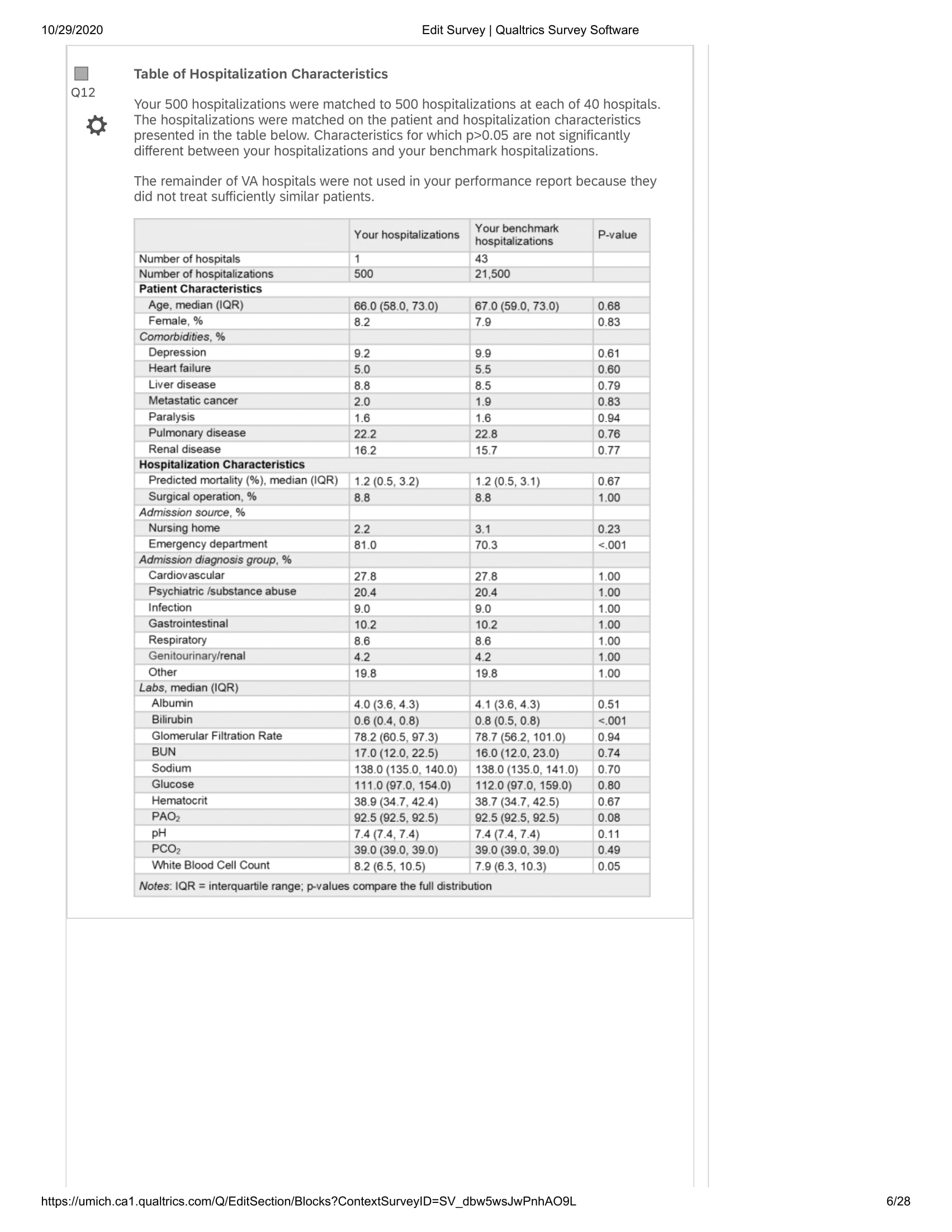


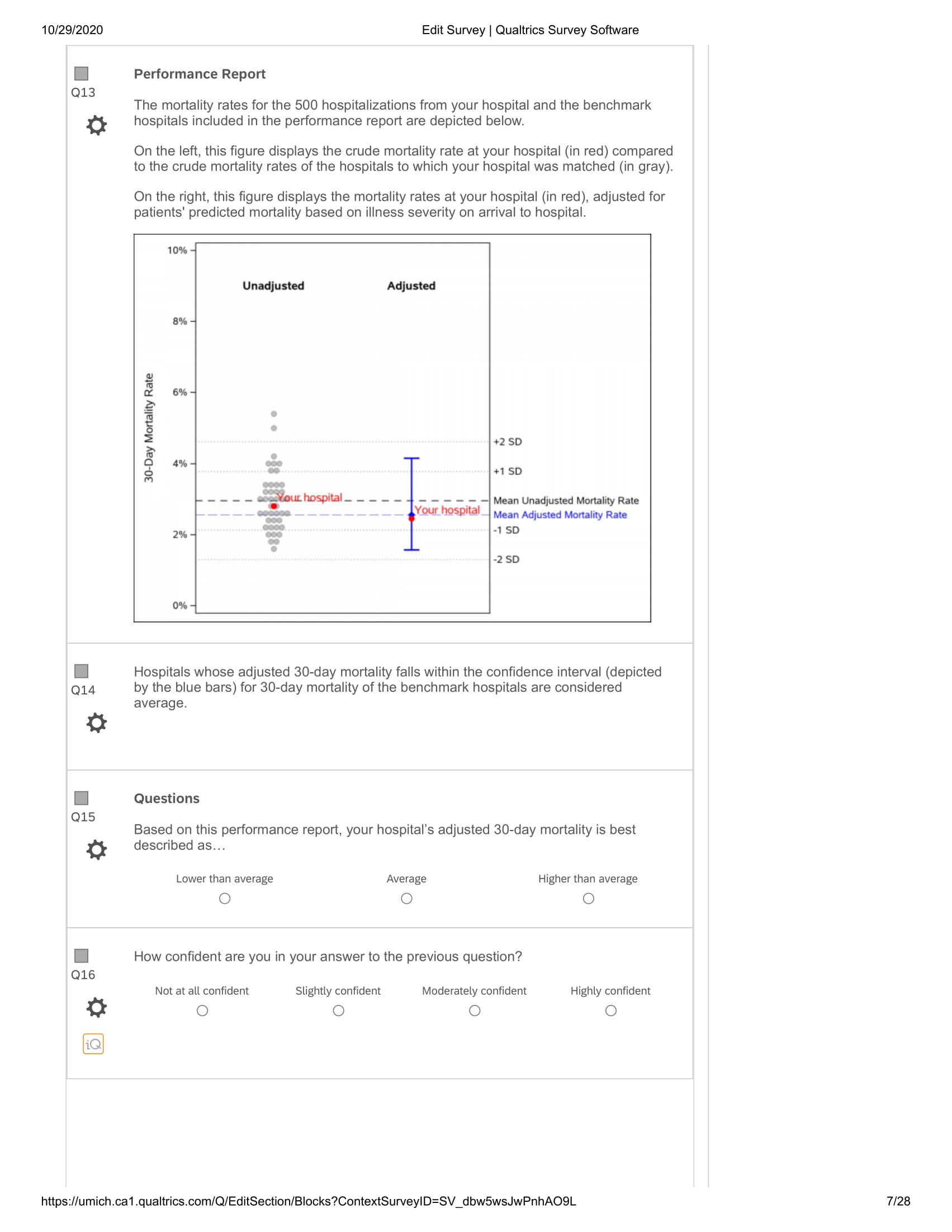


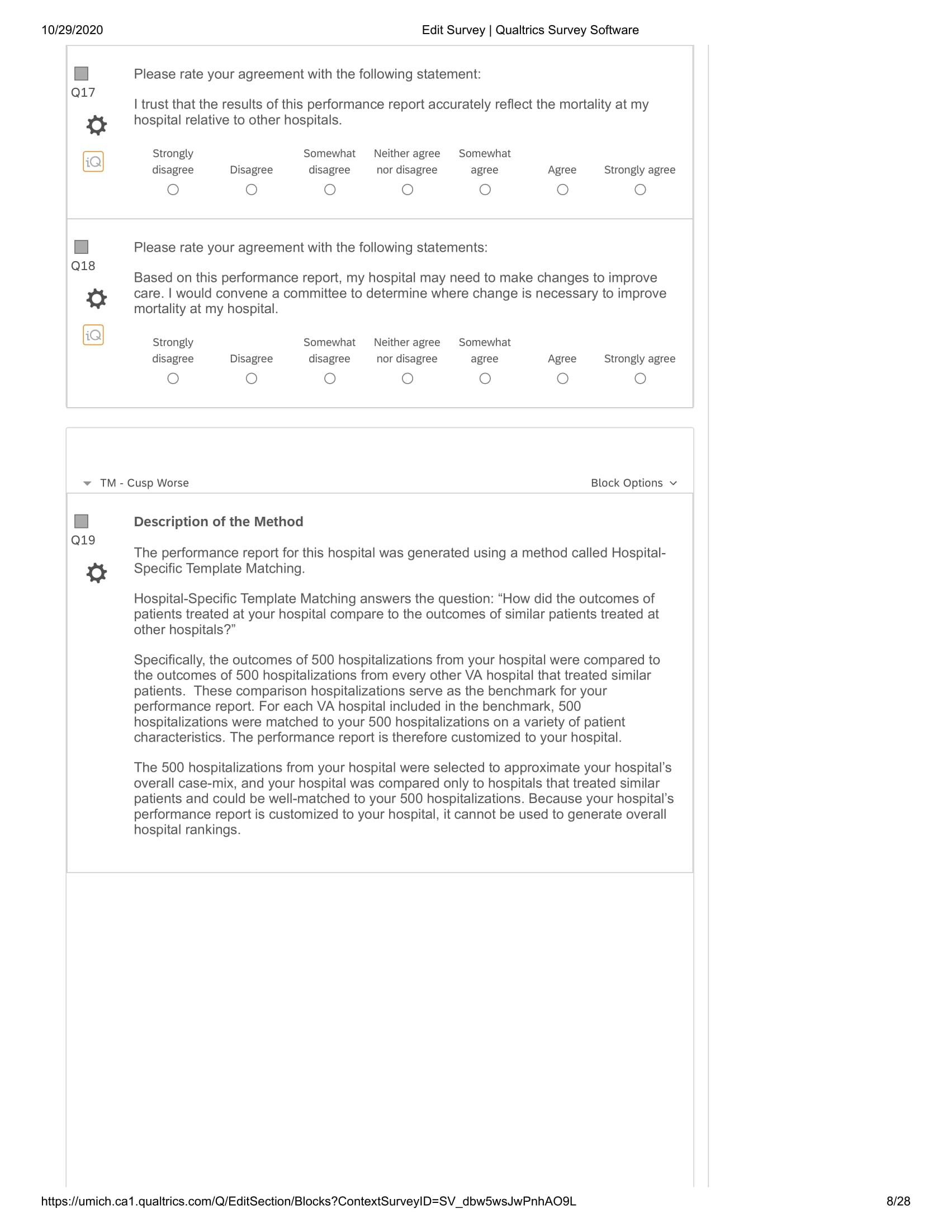


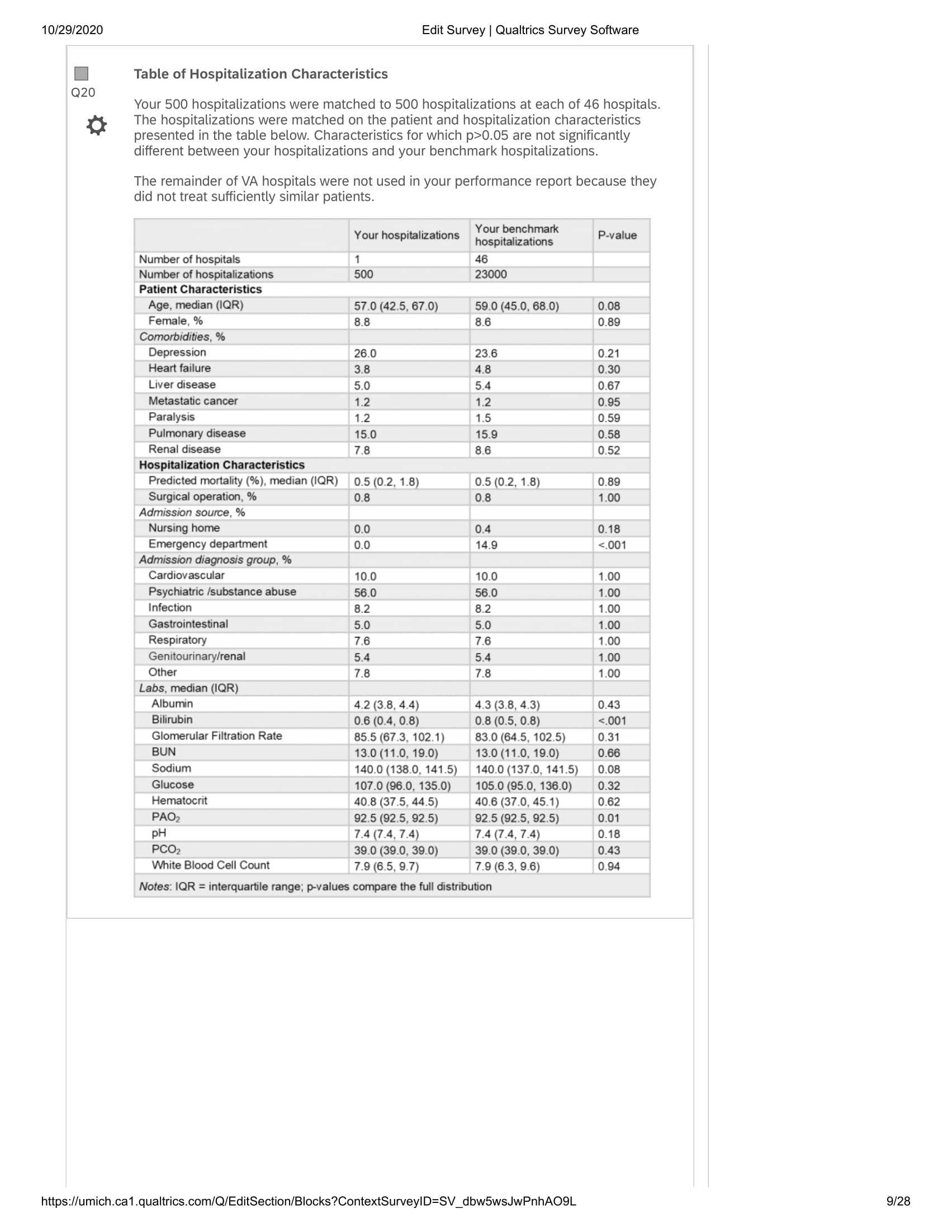


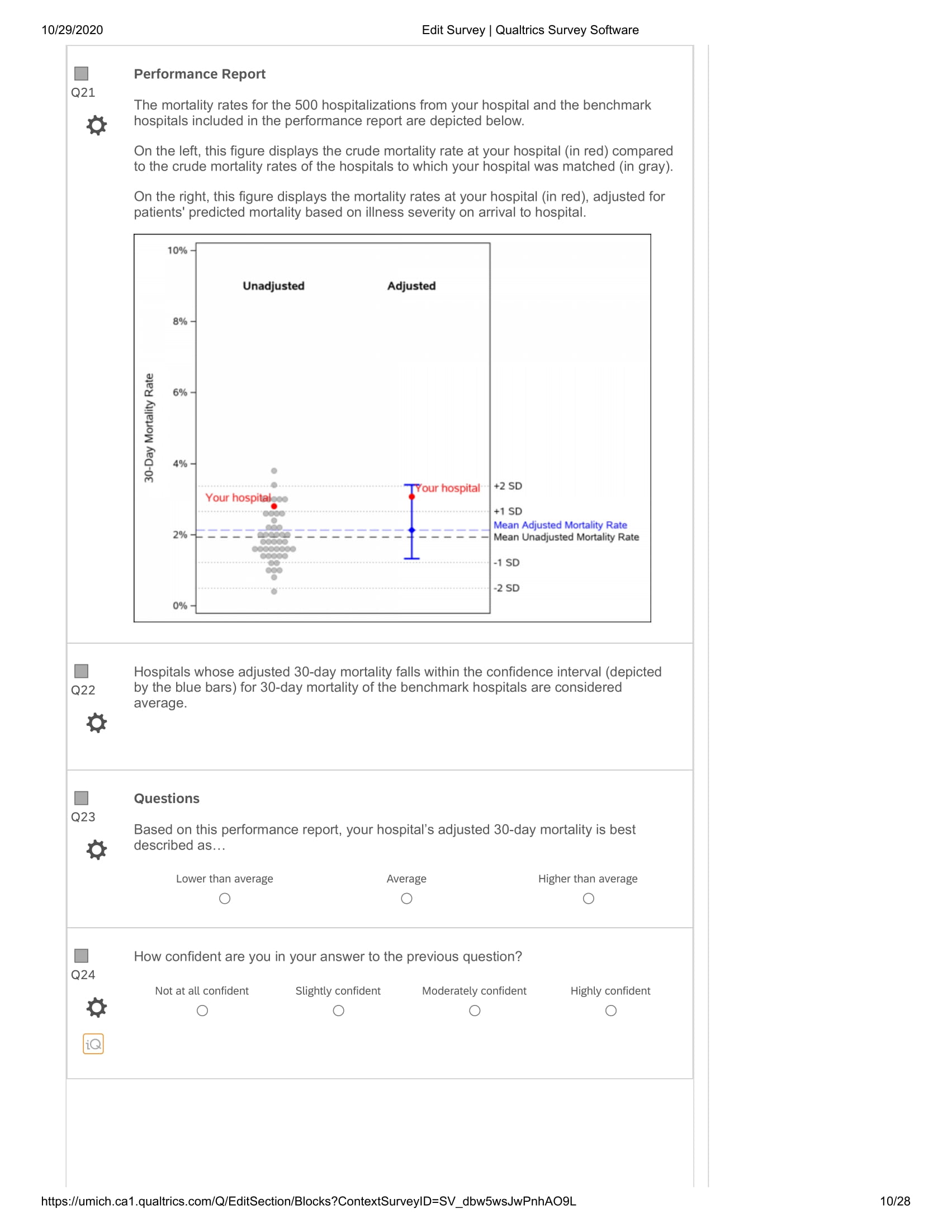


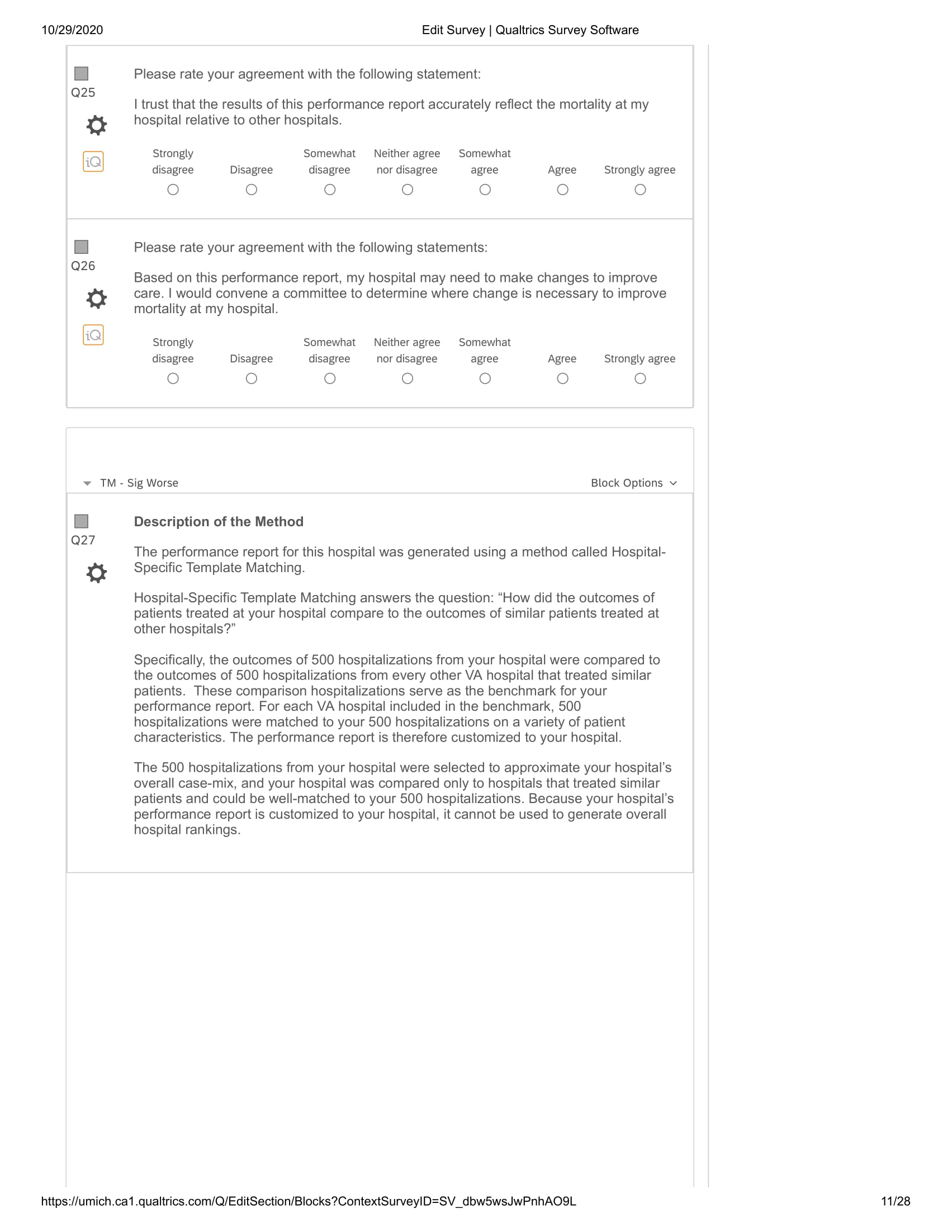


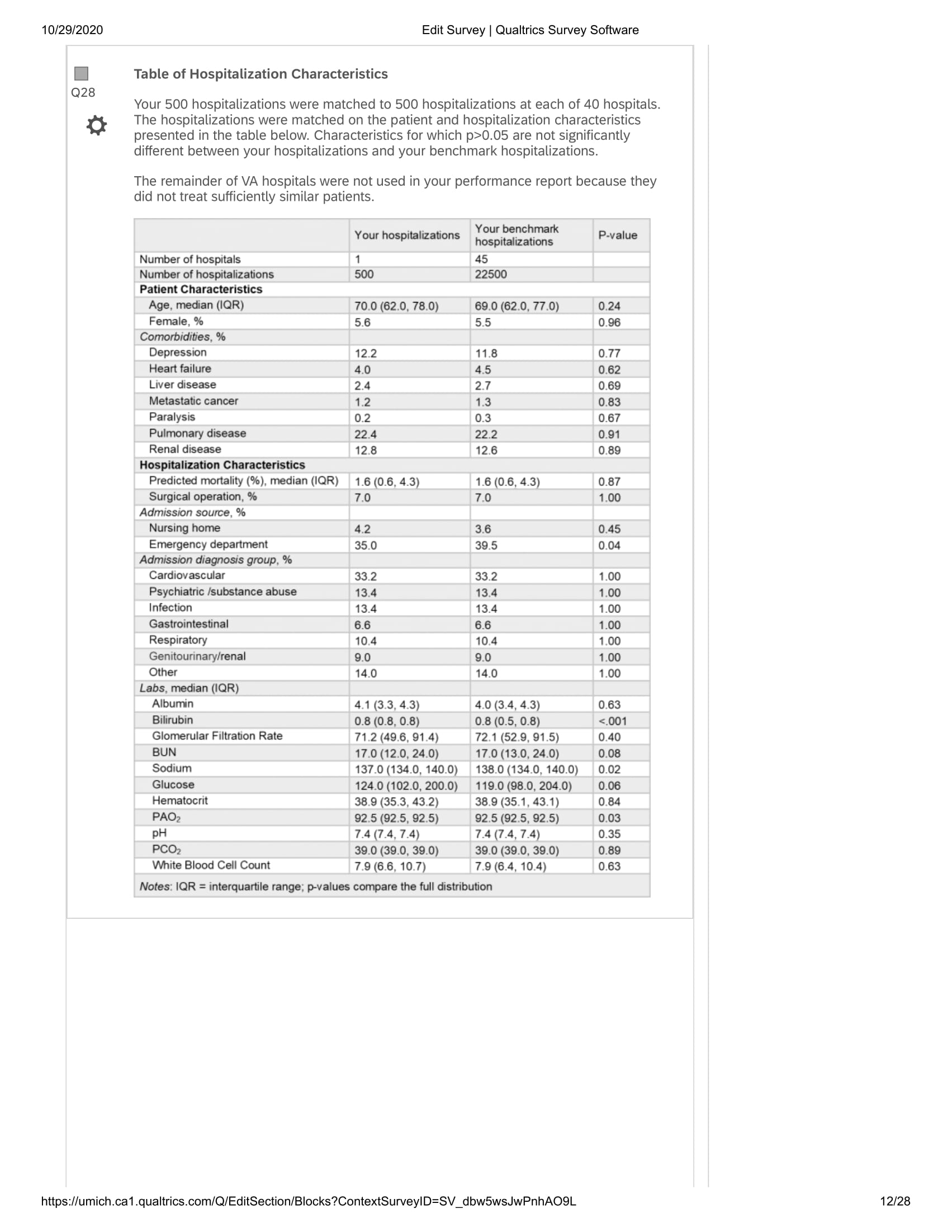


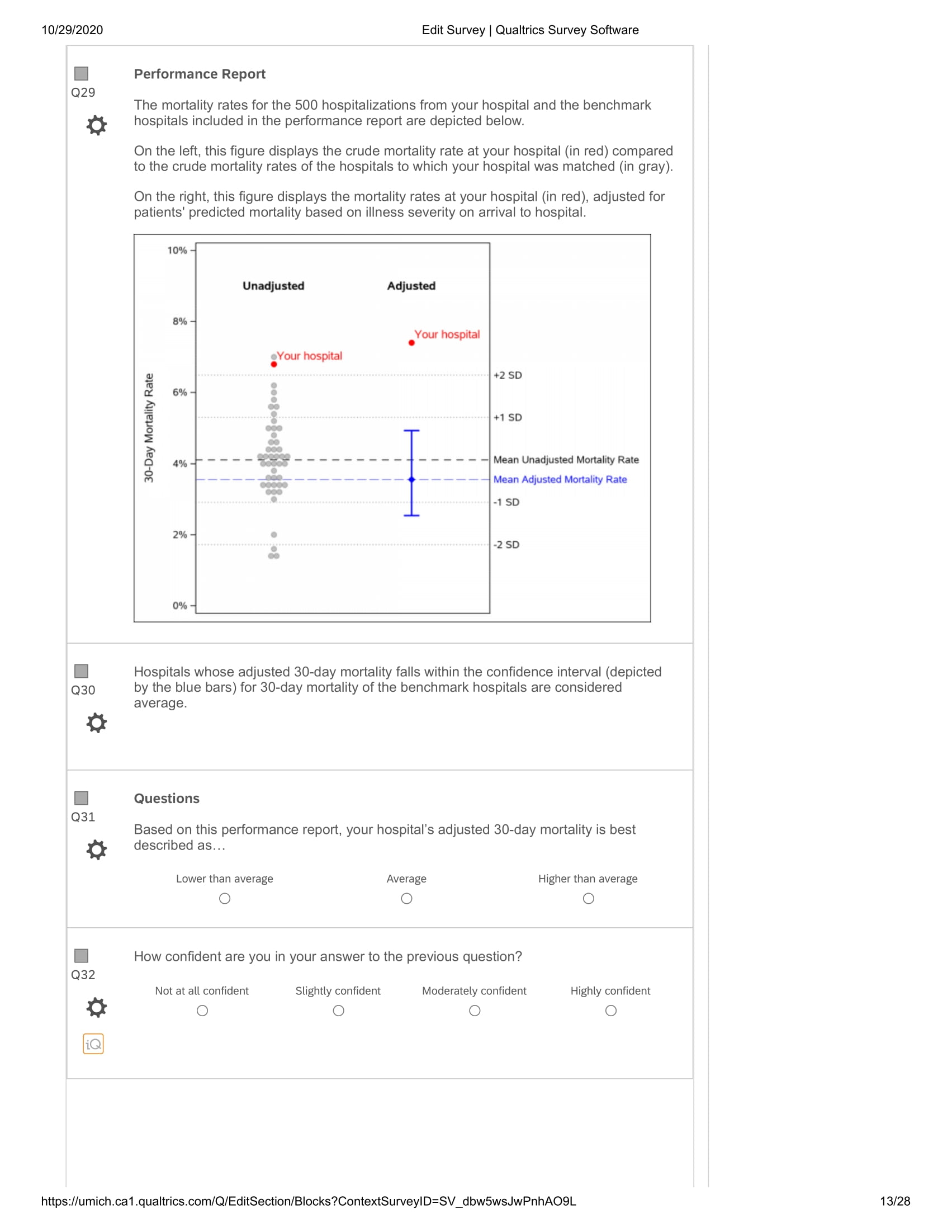


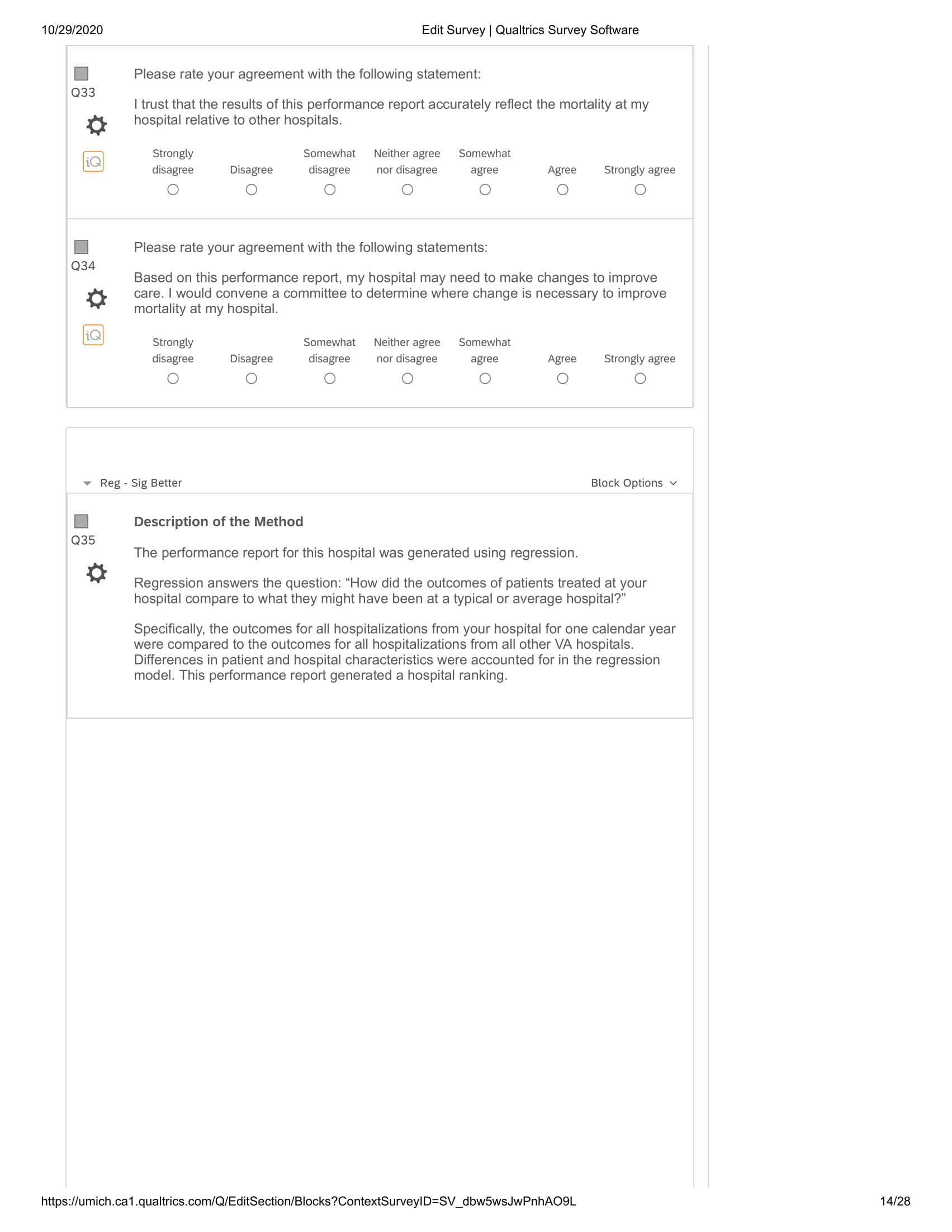


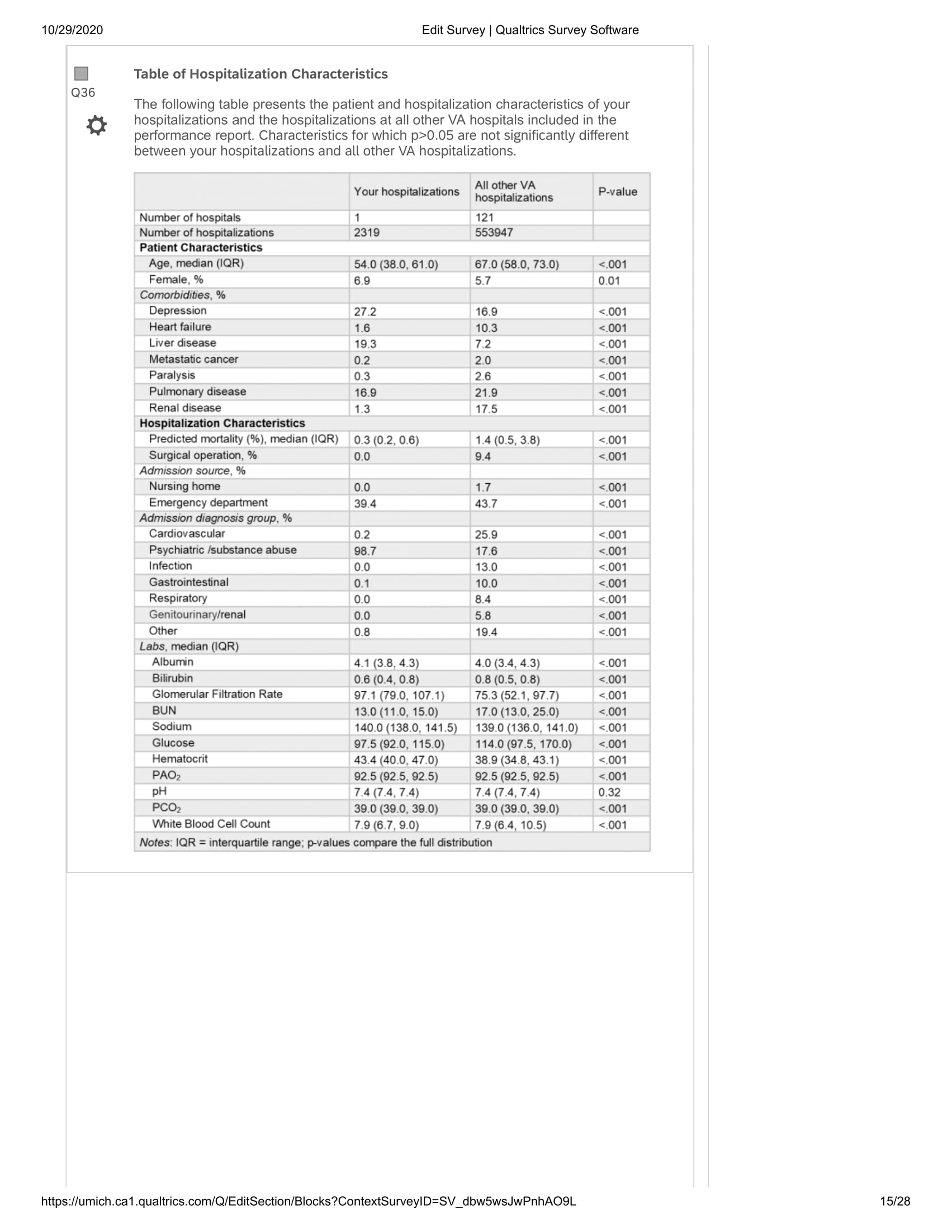


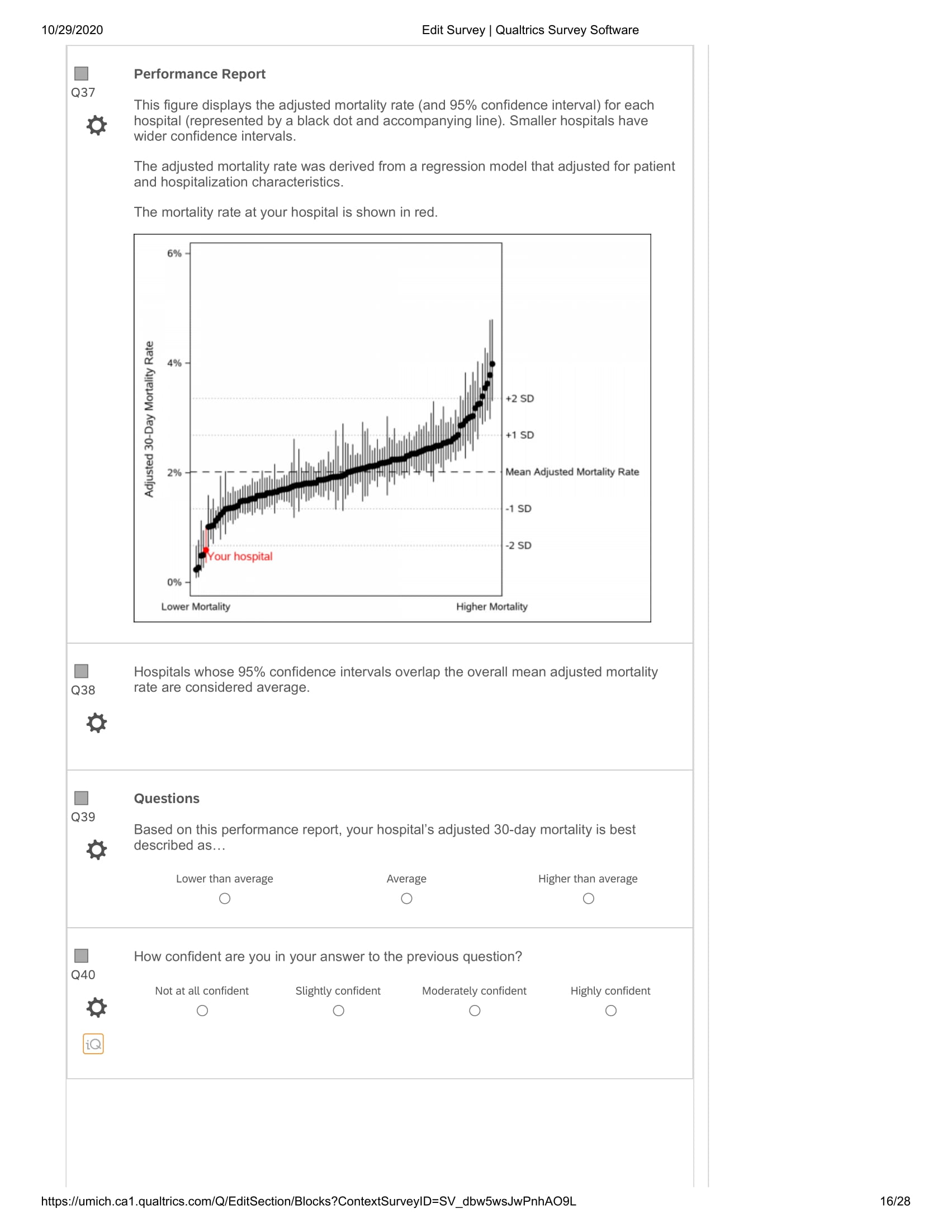


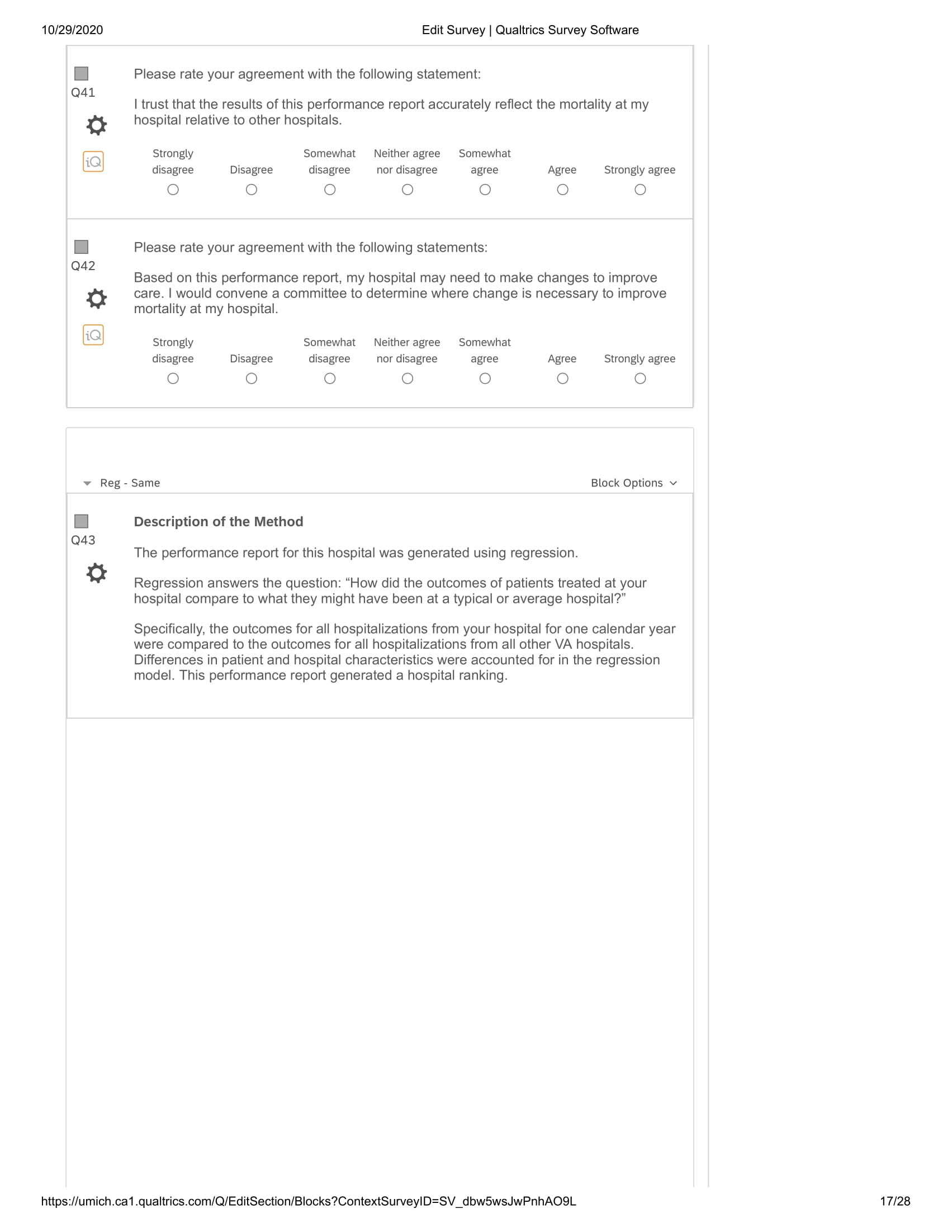


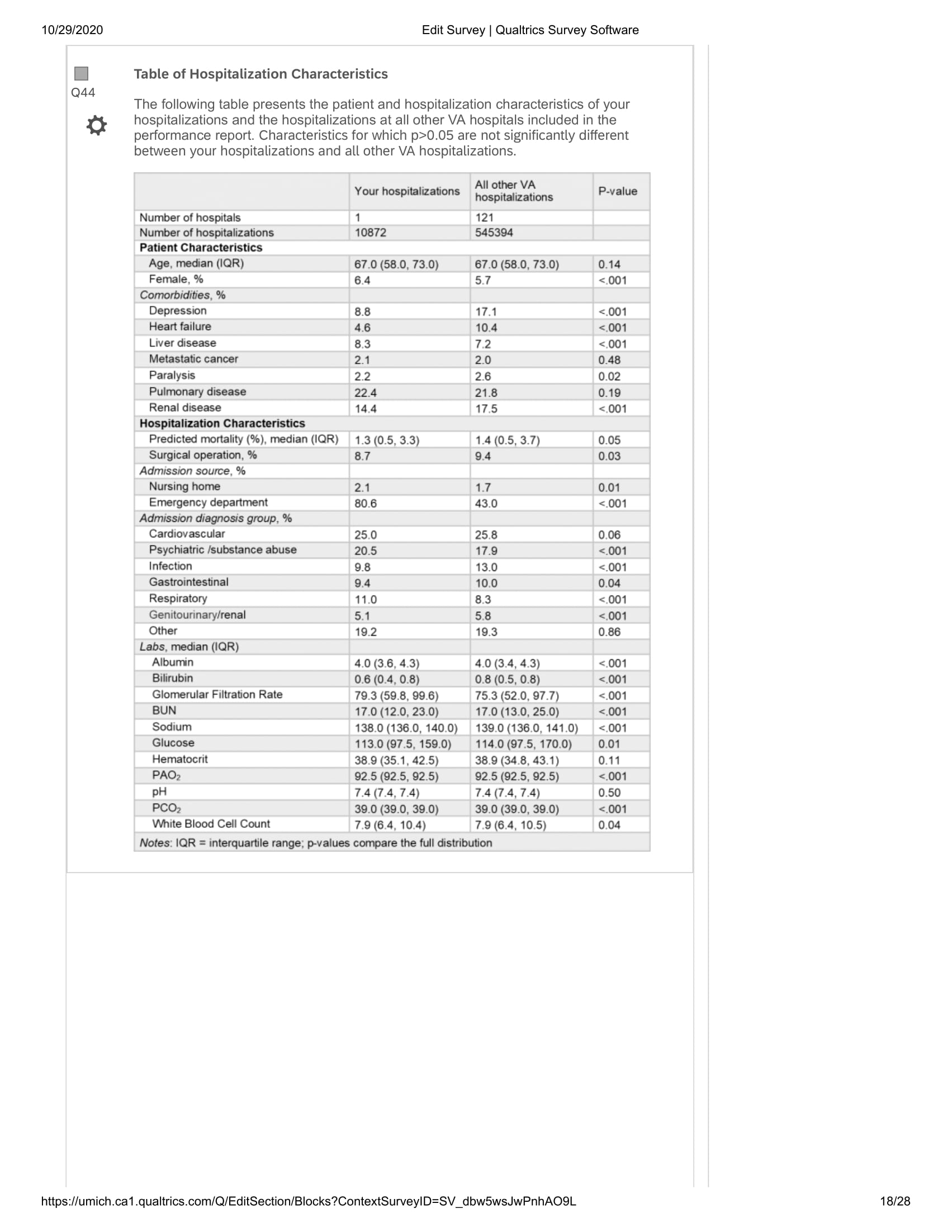


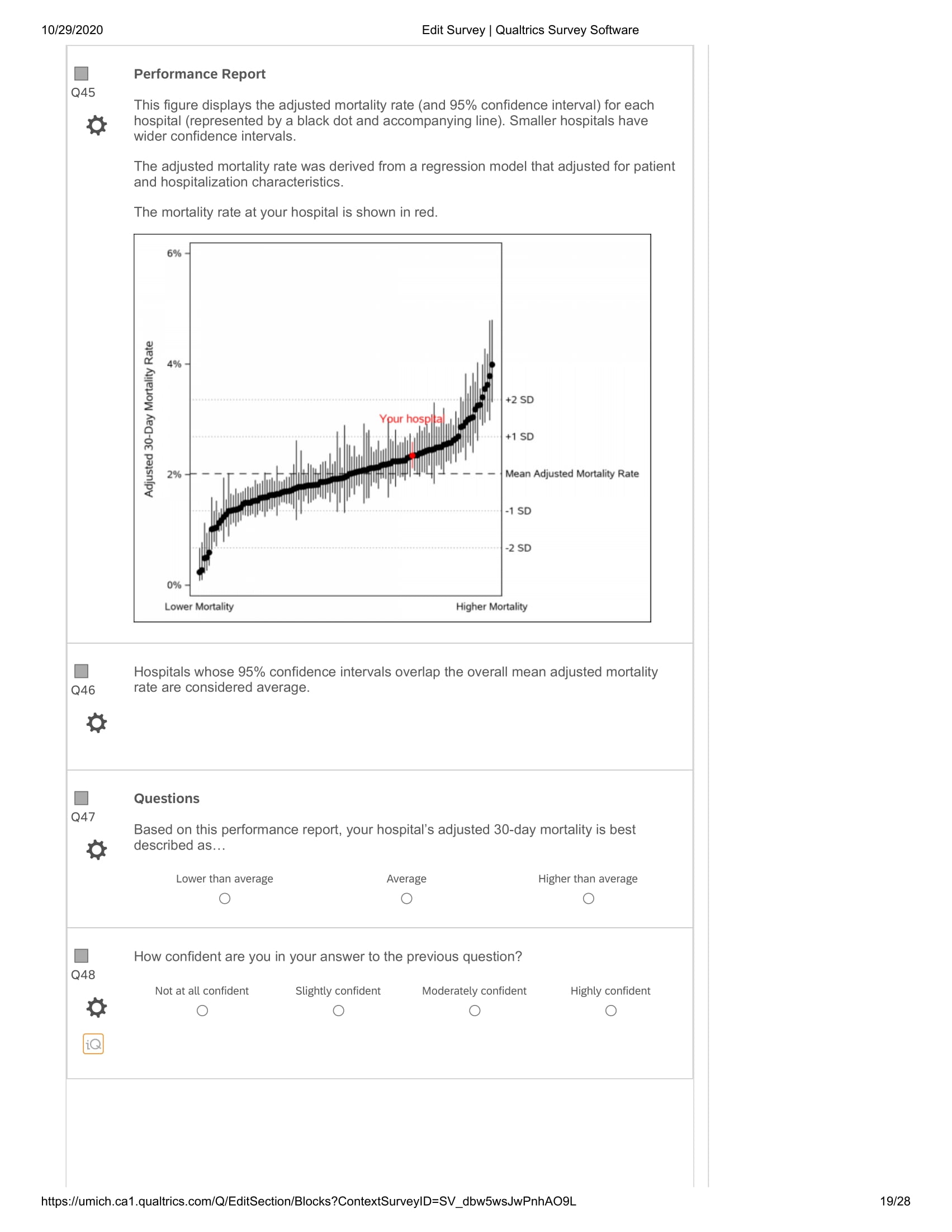


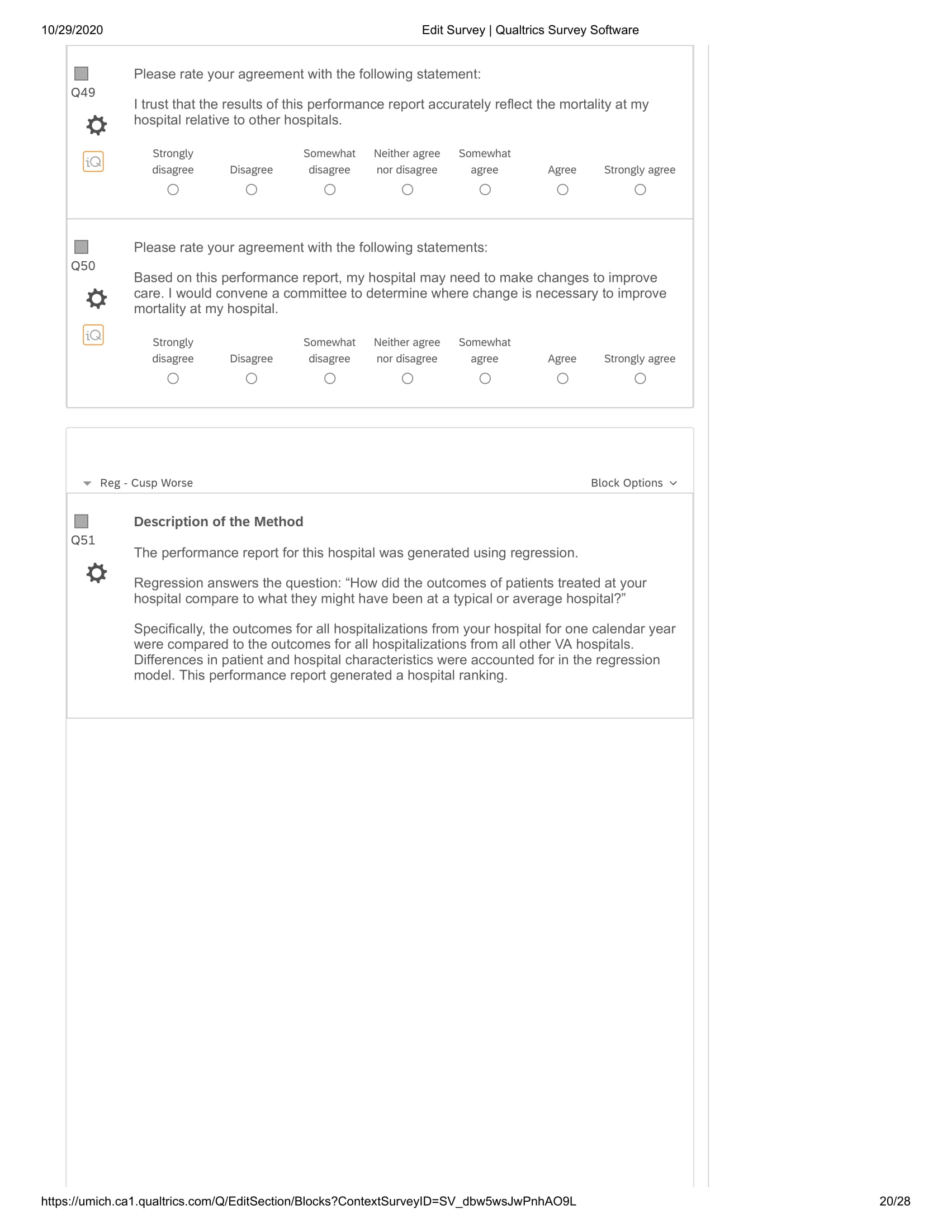


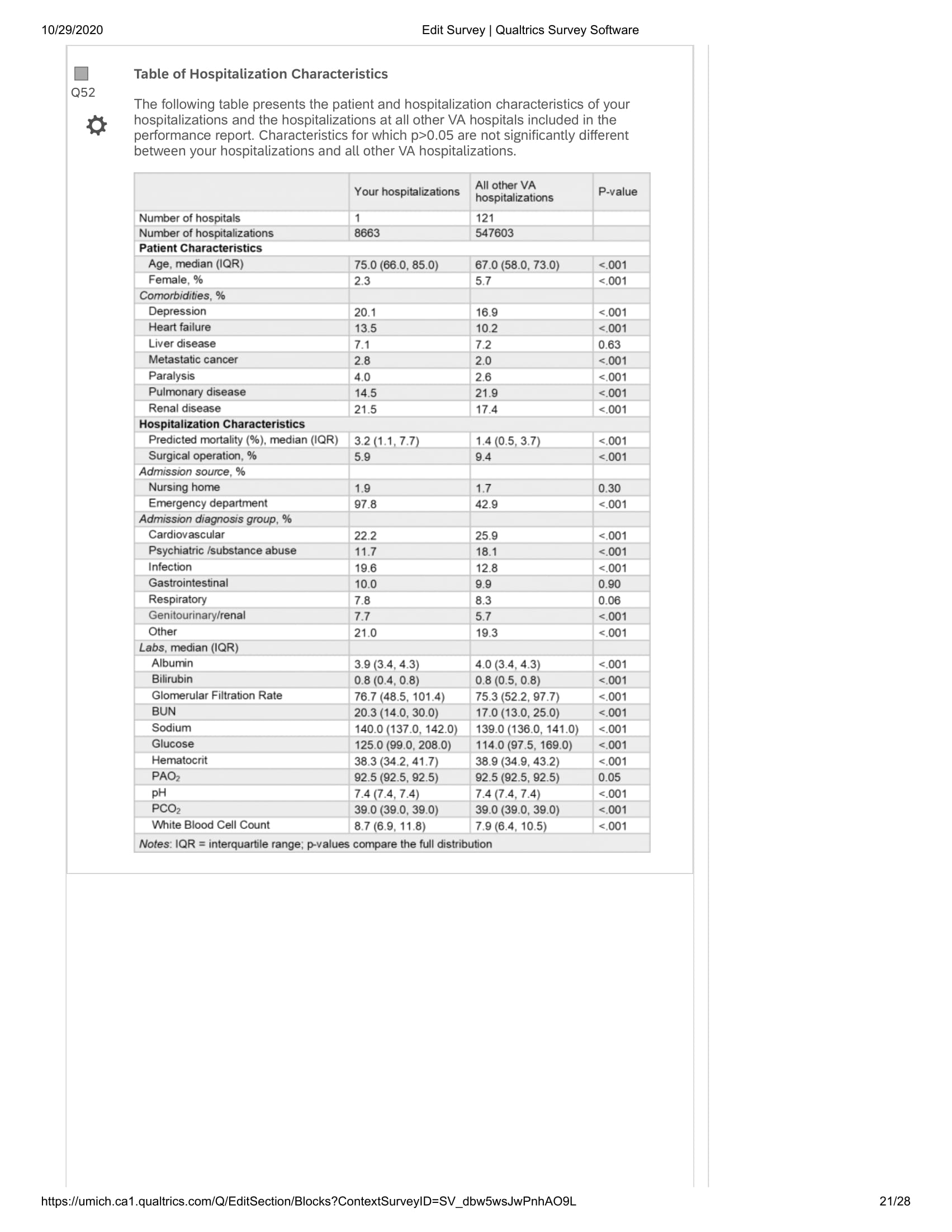


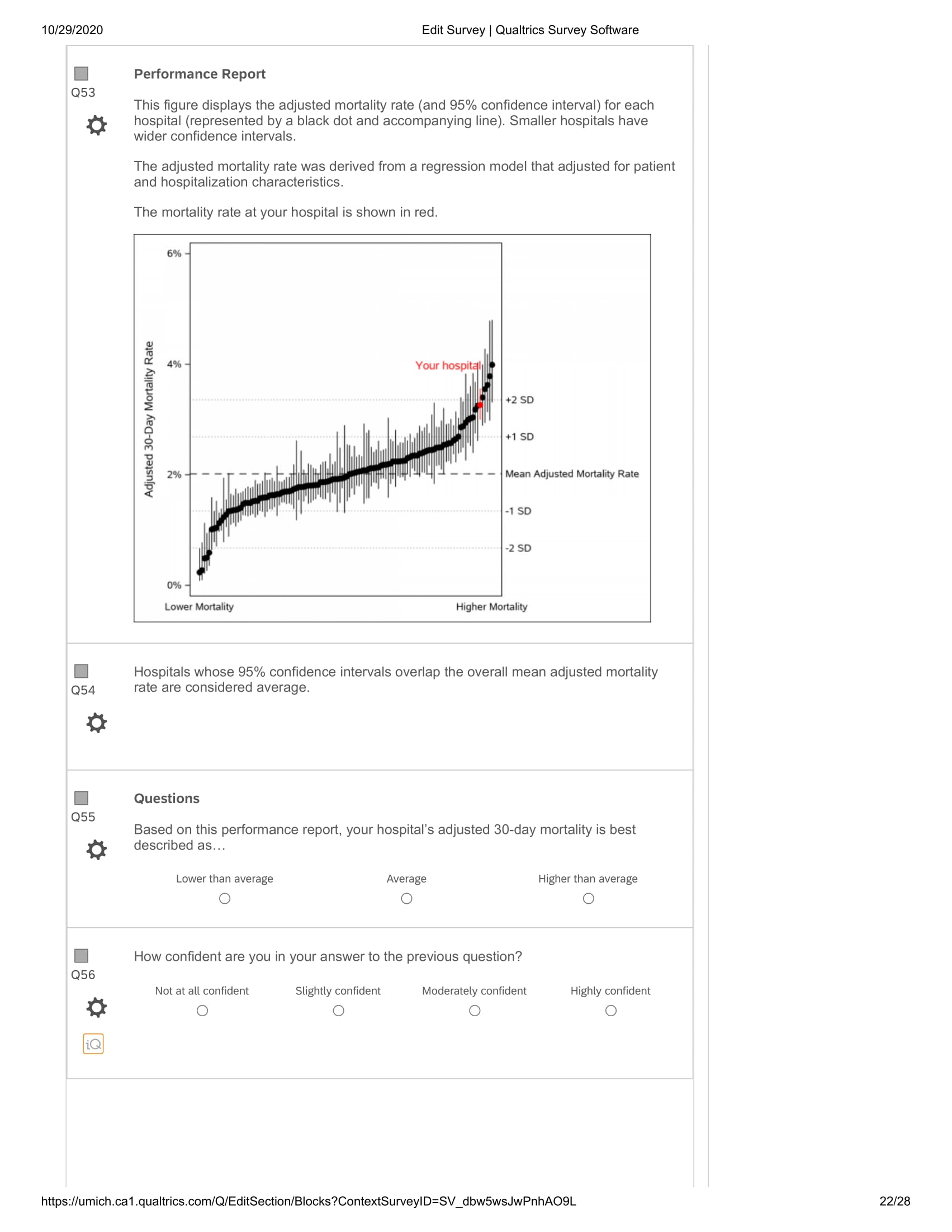


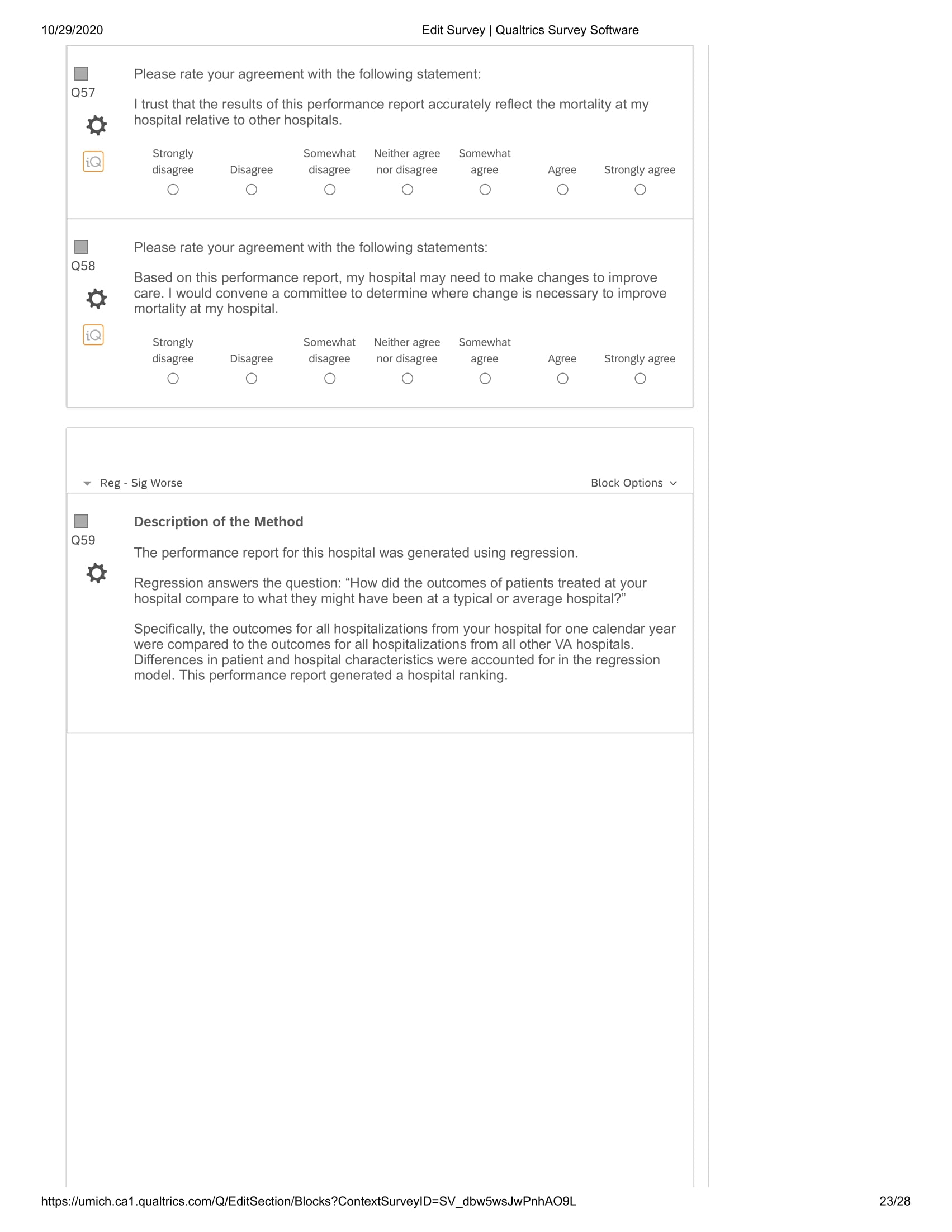


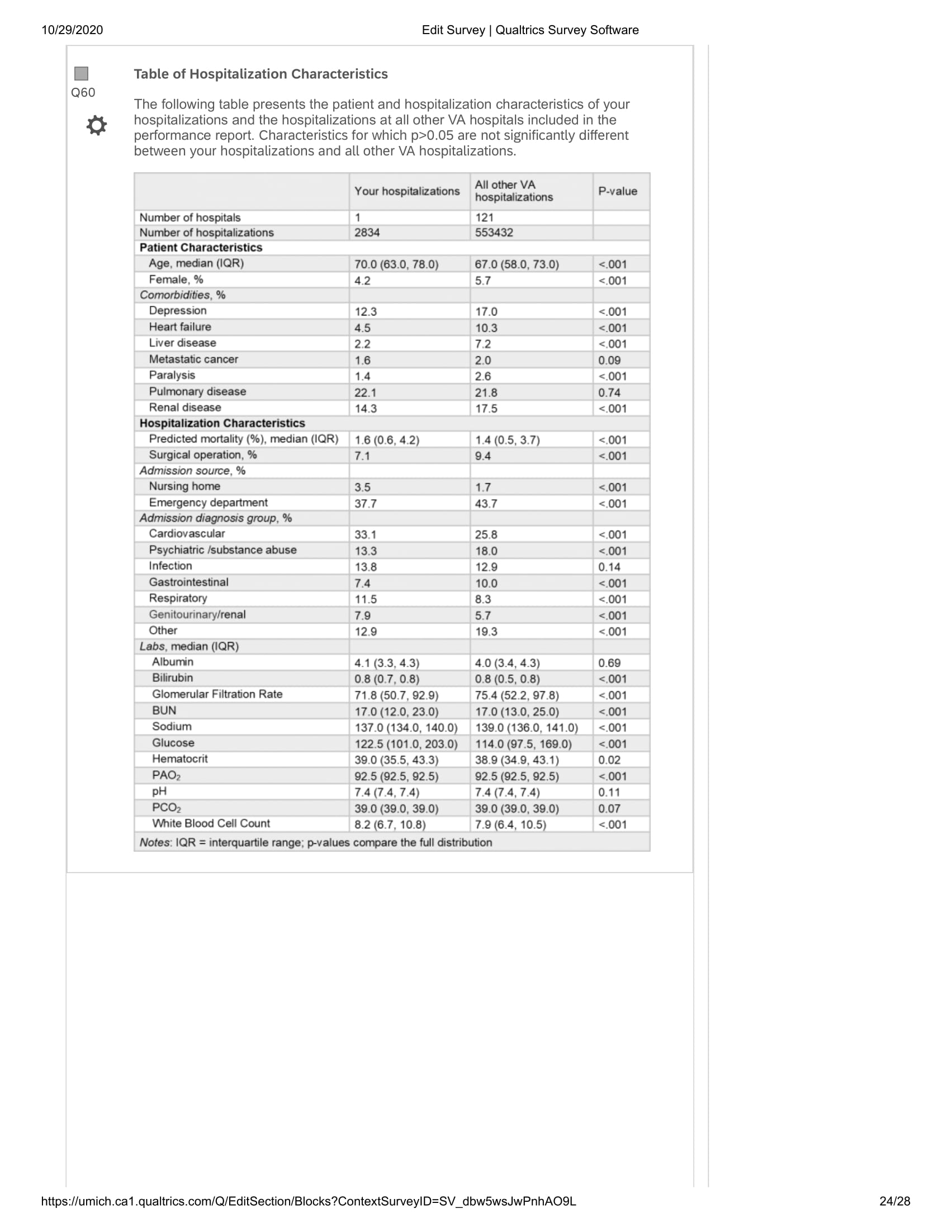


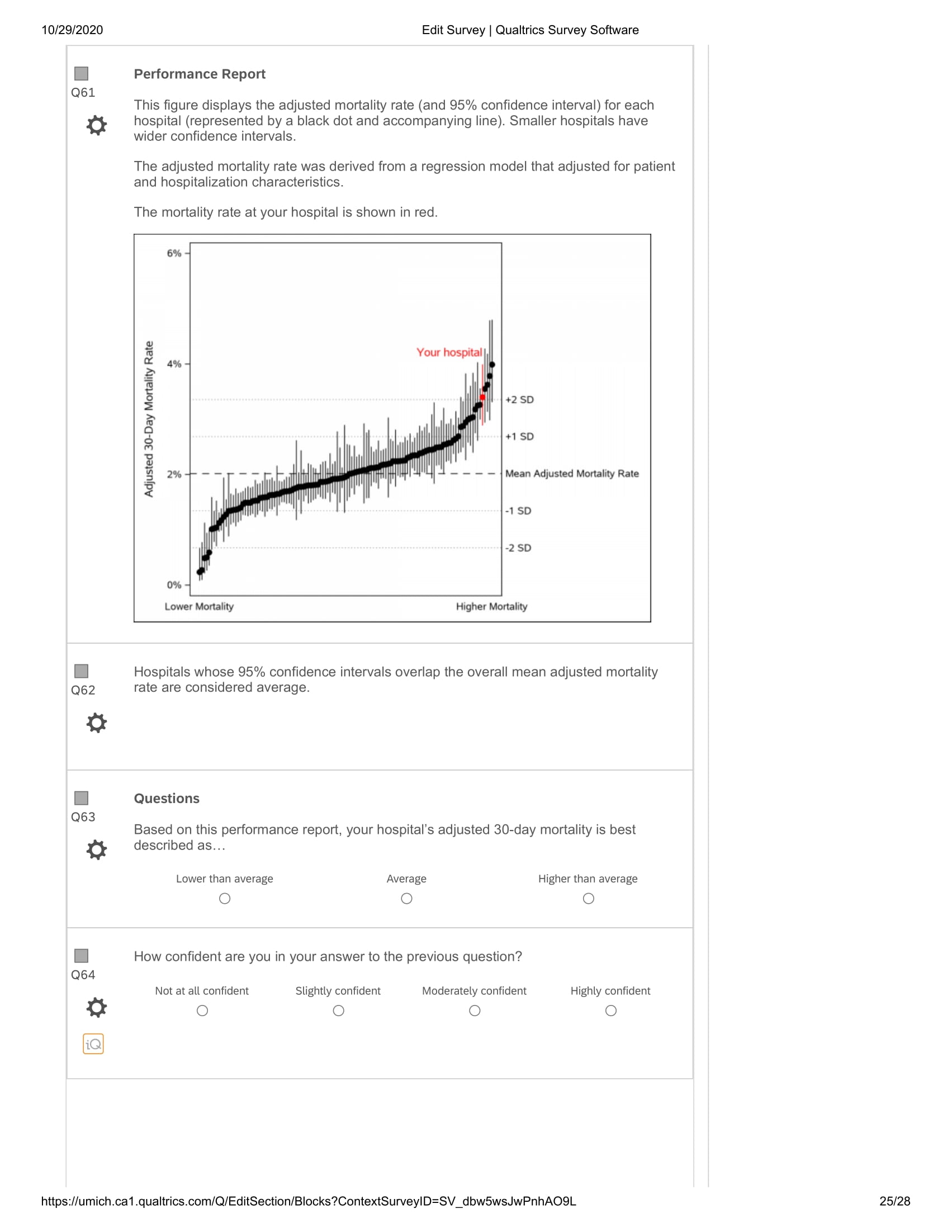


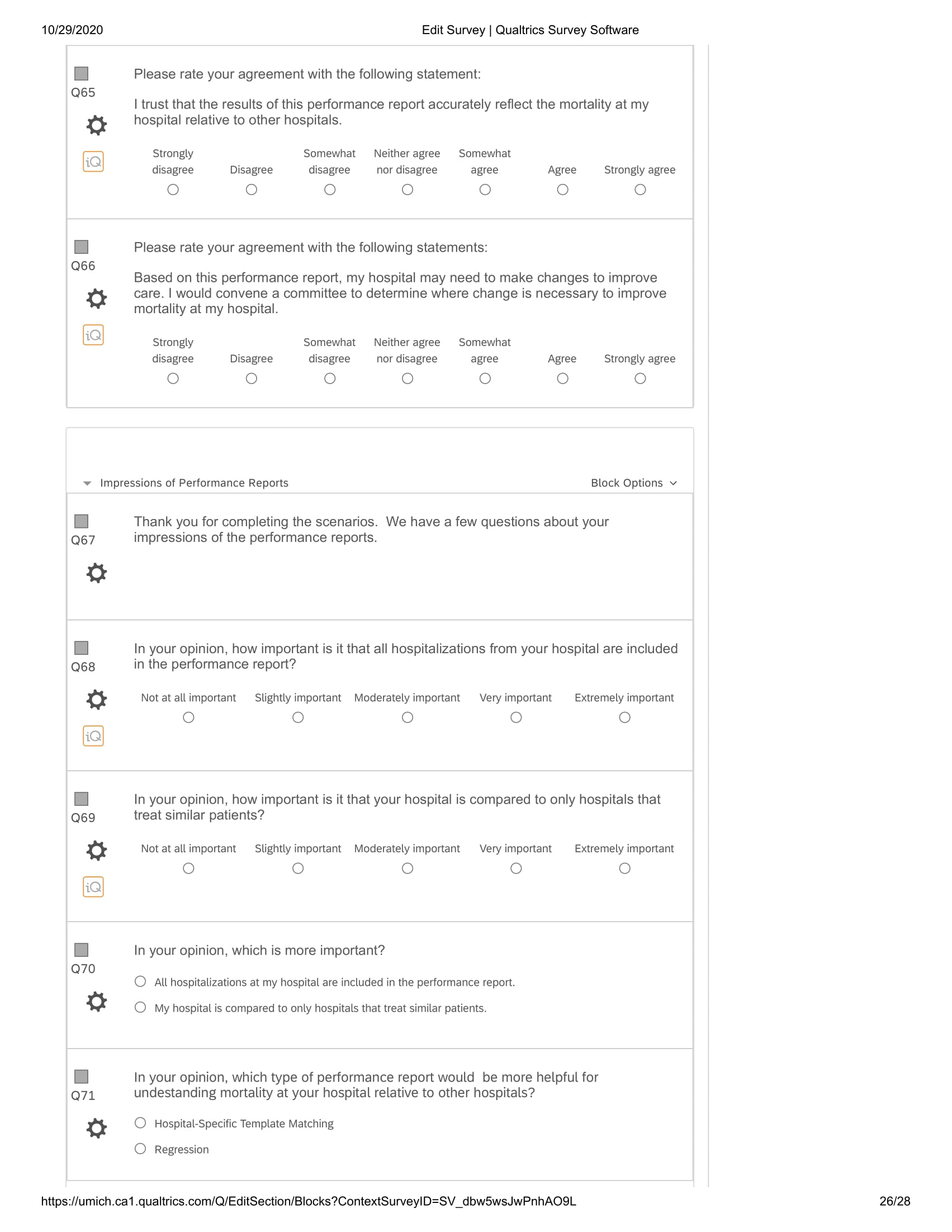


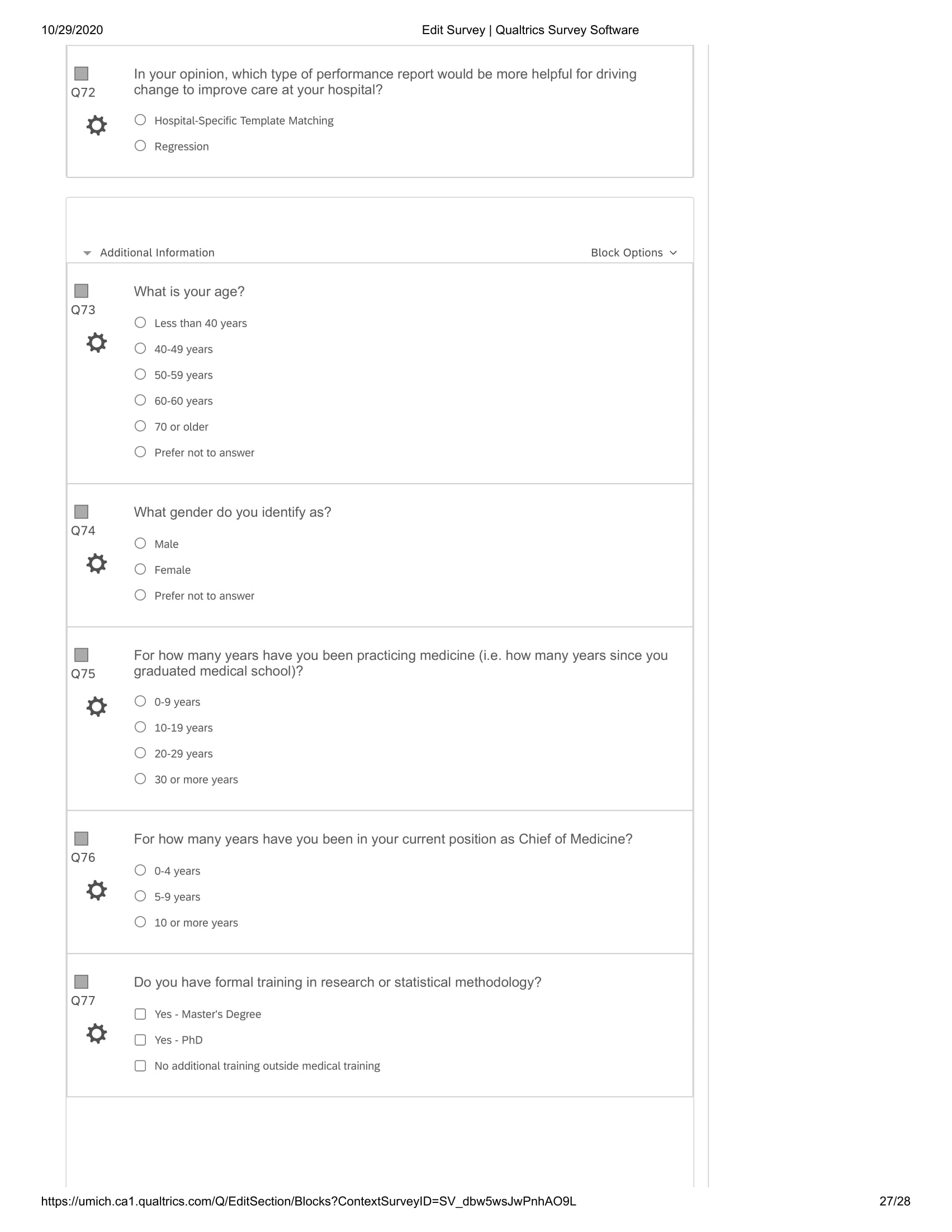


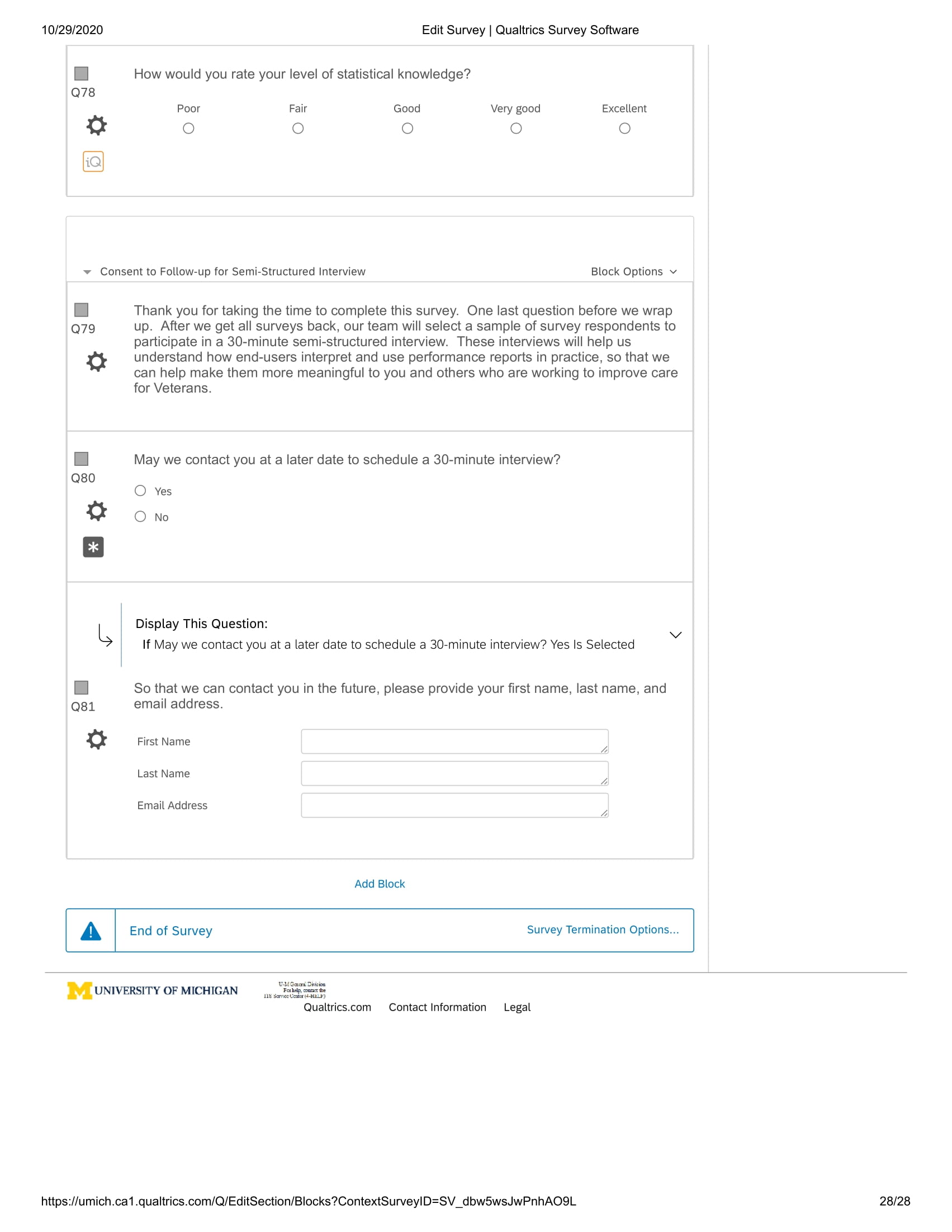


**Appendix 2:** Interview Guide for Semi-Structured Interviews

There were two versions of the interview guide. In one version (as copied below), “Scenario A” asks about the Hospital Specific Template Matching performance report first. In the other version, “Scenario A” asks about the Regression performance report first. The study team used the version of the guide that matched the order in which the participant received the methods in their survey.

**Interview Guide for Semi-Structured Interview with Chiefs of Medicine**

Hello, Dr. _____________, this is ___________. Thank you for agreeing to talk with us today. We also have ___________ on the call who will be recording the interview.

We are talking to you today because we want to learn how hospital administrators respond to different formats for hospital performance assessment. We are not looking for right or wrong answers; instead, we will use this information to make hospital performance reports as useful as possible to administrators.

We would like to record the interview to make sure we capture everything you say accurately. Would this be OK?

During the interview, please let me know if you do not want to answer any question and I will skip to the next question.  You can also ask me to pause the recorder or stop the interview at any time. You also have the right to withdraw from the interview. This interview is voluntary, and your comments will be kept confidential. The interview will be transcribed, and any identifying information will be removed. Do you have any questions for me before we begin?

I’m going to turn the recorder on now and state some initial comments. I will then ask you for permission to record the interview again so we have your consent on record.

**Interviewer – turn on recording device.**

**State**: Date, time, participant ID

“Do you agree to have your interview audio-recorded?”

**Scenario A**

Just as a reminder, for the survey, you looked at four scenarios that employed two methods for generating performance reports. For this interview, we will be reviewing two of those scenarios. So now, we’re going to look at the first of those two scenarios.

**Part 1: Interpretability**

The first scenario we will be looking at generates a performance report using a method called Hospital-Specific Template Matching.

This performance report is customized to your hospital. We selected 500 hospitalizations that approximate your hospital’s overall case-mix. The outcomes of your 500 hospitalizations were compared to the outcomes of 500 hospitalizations from every other VA hospital that treated similar patients. The comparison hospitalizations serve as the benchmark for your performance report. As you can see in this Table, 500 hospitalizations at your benchmark hospitals were matched to your 500 hospitalizations on a variety of patient characteristics. The table shows how similar the hospitalizations are.

So now, let’s move on to the Template Matching performance report. This performance report looks at mortality rates. In the figure on the left, the red dot represents the crude mortality rate at your hospital. The grey dots are the crude mortality rates of the hospitals to which your hospital was matched.

In the figure on the right, the red dot represents the adjusted 30-day mortality rates at your hospital, adjusted for patients' predicted mortality based on illness severity on arrival to hospital. If your red dot falls within the confidence interval, which is depicted by the blue bars, then your hospital is average for 30-day mortality of the hospitals to which you are compared.

1. Could you walk me through how you determined that the hospital was an [under-, average-, high-performer]?

2. What aspects of the method used in this scenario led you to be [confident, non confident] about your interpretation?

**Probes**: What do you think about how the data was presented?

Was it easy/difficult to understand?

**Part 2: Credibility**

3. Was this performance report [effective, not effective] at describing the quality of care provided by the hospital?

**Probes**: Why do you think so?

Do you have concerns with the performance report?

How do you feel about the underlying data?

If you received this report, would you believe it? Would you act based on the information it gives you?

**Part 3: Usability**

4. How might you use the data from this performance report?

5. We are currently using groups of 500 hospitalizations to create these reports, are there other groups would improve usability of the performance report (i.e., ICU vs non-ICU, patients with major surgical procedures, etc.)?

**Scenario B**

Now we’ll move on to the second and final scenario we’ll be reviewing today. To refresh your memory, this scenario presents a performance report generated using regression.

In this method, the outcomes for all hospitalizations from your hospital for one calendar year were compared to the outcomes for all hospitalizations from all other VA hospitals. Differences in patient and hospital characteristics were accounted for in the regression model. This performance report generated a hospital ranking.

In the table, you can see the patient and hospitalization characteristics of your hospitalizations and the hospitalizations at all other VA hospitals included in the performance report. For each characteristic, if p<0.05, then there is a significant difference between your hospitalizations and all other VA hospitalizations.

Now we’re going to move on to the regression performance report.

**Part 1: Interpretability**

In this figure, the black dot and black lines represent the adjusted mortality rate and 95% confidence interval for each hospital. The adjusted mortality rate was derived from a regression model that adjusted for patient and hospitalization characteristics. Your hospital’s mortality rate is shown in red.

1. Could you walk me through how you determined that the hospital was an [under-, average-, high-performer]?

2. What aspects of the method used in this scenario led you to be [confident, non confident] about your interpretation?

**Probes**: What do you think about how the data was presented?

Was it easy/difficult to understand?

**Part 2: Credibility**

3. Was this performance report [effective, not effective] at describing the quality of care provided by the hospital?

**Probes**: Why do you think so?

Do you have concerns with the performance report?

How do you feel about the underlying data?

If you received this report, would you believe it? Would you act based on the information it gives you?

**Part 3: Usability**

4. How might you use the data from this performance report?

5. Are there any other outcomes besides mortality that you’d find useful? [If a lot] How about your Top 3?

**General Questions**

1. Which method did you prefer, Template Matching vs. Regression, and why?

2. Would you use performance reports differently if they were in the form of Template Matching as compared to the current reports you receive using regression? Why?

3. Could you tell me how you use IPEC [In-Patient Evaluation Center] performance reports in your job as Chief of Medicine currently?

**Probe:** Do you find current performance reports useful or not useful? Why is that?

**Probe**: Do performance reports from different benchmarking approaches have different uses? Why or why not? If yes, please describe.

4. What do you think about benchmarking [comparing performance across facilities] in general?

5. Is there anything you would like to add about the two methods that I have not asked?

That is all the questions I have for you today. Thanks very much for your time today. We appreciate you taking the time to help with our research study.

| **eTable 2**: Descriptive Characteristics of Survey Respondents | |
| --- | --- |
| **Gender** |  |
| Female | 21 (30.0) |
| Male | 46 (65.7) |
| Declined to Respond | 3 (4.3) |
| **Time Practicing Medicine** |  |
| 0-9 years | 4 (5.8) |
| 10-19 years | 18 (26.1) |
| 20-29 years | 22 (31.9) |
| 30 or more years | 25 (36.2) |
| **Time In Current Role** |  |
| 0-4 years | 37 (52.9) |
| 5-9 years | 19 (27.1) |
| 10 or more years | 14 (20.0) |
| **Self-Rating of Statistical Knowledge** |  |
| Excellent | 2 (2.9) |
| Very good | 6 (8.6) |
| Good | 24 (34.3) |
| Fair | 30 (42.9) |
| Poor | 8 (11.4) |
| All results are presented as numbers (%). Only respondents that completed the full survey and answered the demographic questions (70/84) are represented here. An additional 14 responded completed at least 1 vignette but stopped the survey prior to completing the demographic questions. | |

| **eTable 3** Survey results for HS-TM-based vs regression-based performance assessment vignettes, stratified by mortality category | | | | |
| --- | --- | --- | --- | --- |
| **Mortality Category** | **Survey Response** | **HS-TM** | **Regression** | **p-value** |
| Below-Average Mortality | Correct ranking | 36 | 33 | 0.36 |
|  | Incorrect ranking | 1 | 4 |  |
|  | Confident in response | 31 | 29 | 0.55 |
|  | Not confident in response | 6 | 8 |  |
|  | Trust accurately portrays mortality | 24 | 19 | 0.35 |
|  | Do not trust accurately portrays mortality | 13 | 18 |  |
|  | Would act | 4 | 6 | 0.50 |
|  | Would not act | 33 | 31 |  |
| Average  Mortality | Correct ranking | 29 | 12 | <0.01 |
|  | Incorrect ranking | 10 | 26 |  |
|  | Confident in response | 33 | 30 | 0.52 |
|  | Not confident in response | 6 | 8 |  |
|  | Trust accurately portrays mortality | 29 | 21 | 0.08 |
|  | Do not trust accurately portrays mortality | 10 | 17 |  |
|  | Would act | 12 | 21 | 0.03 |
|  | Would not act | 27 | 17 |  |
| High-Average Mortality | Correct ranking | 21 | 9 | <0.01 |
|  | Incorrect ranking | 15 | 30 |  |
|  | Confident in response | 30 | 32 | 0.88 |
|  | Not confident in response | 6 | 7 |  |
|  | Trust accurately portrays mortality | 24 | 26 | 1.00 |
|  | Do not trust accurately portrays mortality | 12 | 13 |  |
|  | Would act | 21 | 25 | 0.61 |
|  | Would not act | 15 | 14 |  |
| Above-Average Mortality | Correct ranking | 34 | 29 | 0.42 |
|  | Incorrect ranking | 2 | 4 |  |
|  | Confident in response | 35 | 24 | <0.01 |
|  | Not confident in response | 1 | 9 |  |
|  | Trust accurately portrays mortality | 28 | 21 | 0.20 |
|  | Do not trust accurately portrays mortality | 8 | 12 |  |
|  | Would act | 32 | 26 | 0.25 |
|  | Would not act | 4 | 7 |  |

**eFigure 1.** Interpretabity of HS-TM-based vs regression-based performance assessment vignettes, stratified by mortality category


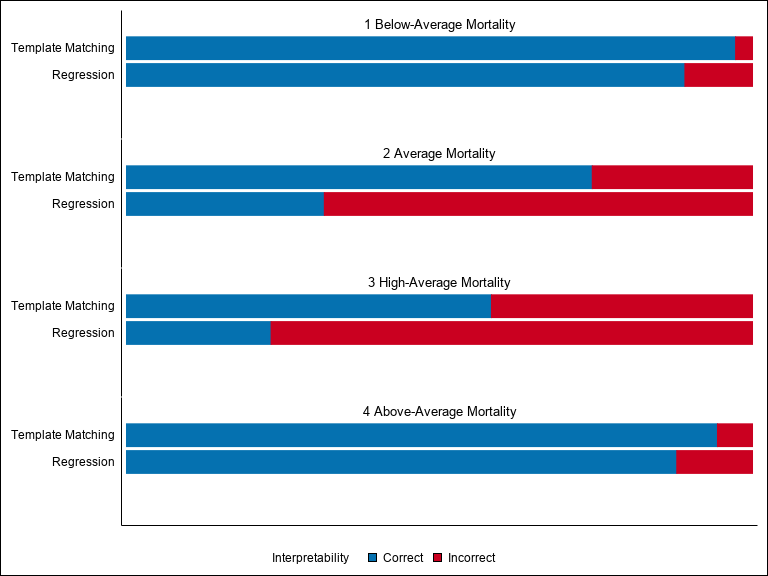


This figure shows the proportion of respondents that correctly identified the 30-day mortality as lower than average, average, or higher than average.

**eFigure 2.** Trust in HS-TM-based vs regression-based performance assessment vignettes, stratified by mortality category

**
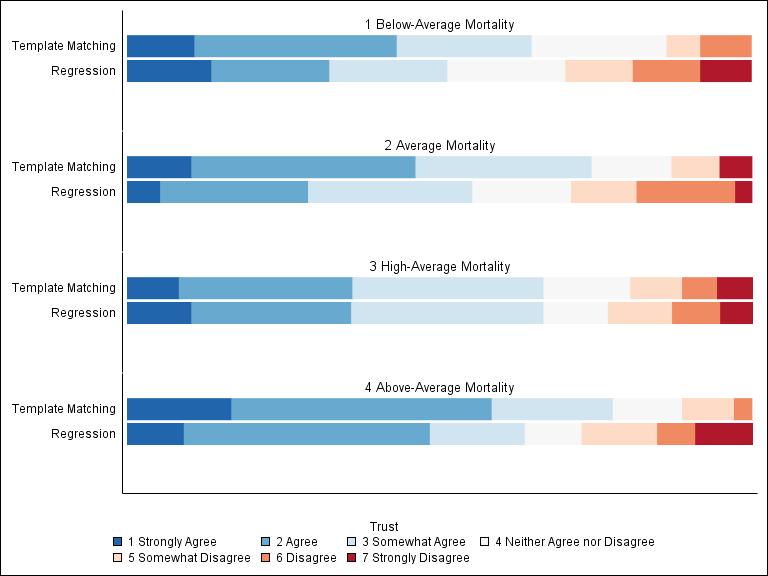
**

This figure shows responses to the survey question: “Rate your agreement with the following statement: I trust that the results of this performance report accurately reflect the mortality at my hospital relative to other hospitals.”

**Appendix 3**: Summary of interview responses regarding fairness and credibility

*Documentation and capture of case-mix/illness severity*

For both methods, most participants expressed concerns about how accurately clinicians are documenting, and how well that is captured in coding (TM01, 03, 05, 07, 09, 11), and whether they accurately capture case mix or illness severity TM01, 09, 10). Several said that performance reports may not depict quality issues; instead, they may be the result of the way the inclusion criteria were defined rather than an actual problem (TM03), or that documentation of comorbidities is a driver of performance (TM09). One respondent often questions the adjusted mortality rate because of case severity index, which is dependent on coding (TM11). Another found that their admission diagnoses were incorrect, so that despite their low acuity level, they weren't meeting standards (TM05). Others make sure to verify periodically whether clinicians are documenting correctly (TM01) and compares their case severity to peer hospitals to see if they are capturing things properly (TM11). Finally, a participant pointed out that there may be differences in how documentation occurs between hospital*s,* e.g., whether one hospital is using trainees versus hospitals that have documentation specialists who are very involved in the charting **(**TM07).

*Accounting for special populations and referral center*

One participant expressed concerns over whether the performance assessment can account for the fact that the participant’s hospital is a transplant center, a heart failure center, a cancer center, has an active palliative care group (TM08), while another said regarding differences in facility complexity, “adjusting for all these different variables, I think that becomes more difficult, the more differences there are” (TM07). Participants expressed similar concerns about the TM model’s ability to account for facility complexity (TM11). Even if matched hospitals have similar proportion of patients with cardiovascular, psychiatric, infection, or GI issues, one participant’s hospital serves as a referral site for other hospitals and will therefore treat more complex patients within those groupings (TM10). A participant at a transplant center was concerned about adjusting for case-mix at the extremes because there might be exclusions that might bias against those centers (TM08). Geographic area was another concern for TM model (TM03, TM07), as well as racial mix (TM03).

*Hospital rankings*

Participants pointed out that being ranked in lower tiers by regression doesn’t necessarily equate to underperformance (TM03): “when the VA chooses to rank things from you know the top performing hospital to the lowest performing hospital being a low performer in that type of ranking doesn't necessarily mean that you're performing badly" (TM09).

*Opaqueness*

Participants pointed about a need for transparency in metric definitions in the regression model; for example, “[W]e have committed enormous resources to attending to a patient's heart failure issues after discharge, so that they don't get readmitted in 30 days…it doesn't matter if they're readmitted because they stub their toe, it's considered a heart failure readmission. So, all of our efforts to make sure that they don't go back into congestive heart failure may have absolutely no impact on the readmission rate if they're being admitted for things other than another episode of congestive heart failure” (TM05).

*Power, size, and event rate*

Regardless of the method, participants were concerned about small sample size and event rate. For example, a small facility could become an outlier with just one or two events, as with healthcare associated infections such as CLABSI (TM04, TM05, TM09, TM10). Even if a hospital is ranked among lower mortality rates, things can change quickly because even just a few “disastrous things” could swing them in the other direction (TM10). Finally, a participant raised doubts about power of TM model (TM03).

*Mortality*

A common attitude among participants was that while mortality rates are a “tried and true” and “reasonable” metric, they are not “surrogates for quality,” but are “alarm triggers” (TM03, TM09). Participants mentioned several critiques regarding whether comparing hospitals on mortality captures differences in quality. A large urban hospital with a tertiary or coronary care program might be seeing more high-risk cases which would affect mortality rates, and patients may be different even if demographics or diagnoses are the same. One participant said that “the definition used to put people into these bins is actually flawed.” He gave an example of a patient with stage four pancreatic cancer who is hospitalized, and the hospice team is consulted, and the patient moves to the hospice unit and dies two weeks later. But if hospice didn’t see the patient within the first 24 hours, that death gets counted in SMR data (TM03, TM07, TM11). A participant said that comparing “softer metrics” like patient preferences surveys may be more useful because he could see what high satisfaction facilities are doing that can be transferred (TM07). Another participant mentioned important measures besides mortality, which included the length of time people are intubated in ICU, infection rates in different areas, and 7- or 30-day readmission rates (TM10).

*Other*

Other concerns included inclusion/exclusion problems [TM], gaming the system, and the difficulty of defining quality: “it's kind of like that Supreme Court justice said like I can't define it, but…I know it when I see it… if you're a chief of a department and you're worth your salt you definitely know it when you see it, but a lot of poor-quality care slips through the cracks” (TM03). Participants observed that if a facility is in either tail, then it’s likely there is something unique about them that merits attention; however, if they’re closer to the mean, then there could be issues about what’s included in the model or how variables are weighted [regression] (TM04). Concerns were raised over statistical vs clinical significance. The p values in the table are statistically different, but the numbers may be clinically similar due to natural variation in the way testing is performed and different test methodologies in different hospitals. There may be instances in which “a value that is statistically different is well within the range of clinical variability.” [regression] (TM08, TM11). Finally, participants expressed a preference for criterion reference (e.g., 15% readmission rate plus or minus 3) over norm reference (e.g., for wait times, a VA facility might be low ranked in the VA overall, but better than community wait times) (TM11). In addition, with regards to the TM model, hospitalization characteristics do not include socioeconomic issues that have a high correlation to mortality rates (TM08), and there were doubts about its internal and external validity.

**Appendix 4:** Summary of interview responses regarding usability

*Deep dive*

A common sentiment about getting a performance report was that “in and of itself, the data doesn't say you're good, bad, or indifferent” (TM05), but that any report is “a flag or an indicator for something that that we might need to respond to” (TM04) or “a trigger for a deeper dive” or a “red flag” (TM03, TM10). A deeper dive should occur before sending it to clinical staff. In any case, it’s inappropriate to use the reports in a punitive or even positive manner (TM04, TM05, TM07). If the mortality rate is higher than average, participants said they would investigate the driver of mortality. For example, is it coming from a specific service in which people are likely to die? Is there anything unique about their patient population or any specific care-related practices? Who died, why did they die, how did they die? Was it a one-time occurrence that corrects itself? (TM03, TM04, TM05, TM07, TM10). They would check their own data to break down their mortality into disease categories or parts of the hospital, such as ICU vs inpatient mortality, and how they use hospice and acute care (TM01, TM11). Several mentioned checking the face validity of the reports to their own lived experience in hospital leadership and their knowledge of the hospital (TM05, TM08, TM11), or the opposite, in finding value in data that contradicts their “gut check” (TM07). Some would try to find longitudinal trends (TM01, TM11). Some said they needed to determine whether the higher-than-average mortality rate is a quality-of-care issue versus a documentation issue (TM09, TM11), or whether they’re capturing all of the factors that go into the model” such as labs (TM09) and accurately capturing CSI (TM11). One participant mentioned creating rapid response teams after looking at mortality from the codes when they occur and comparing that nationally to how many people are resuscitated successfully in house, which reduced the number of codes (TM10).

*Motivation*

Multiple providers said that if a facility’s mortality rate is higher than the mean, this can serve as motivation to improve processes, that you “get on it with a sense of urgency” (TM01). A provider said that if a facility is an outlier, that data can be used “to impress upon certain stakeholders that this is indeed something that we need to devote some energy to. You know, particularly if you know, we find that there is a certain service line that that seems to be overrepresented in our mortality” [regression] (TM04). However, another provider said that before using the data as motivation, it should be verified first: “I think it makes them more invested in the outcome if they see that this is actually what's going on in their hospital, right? And this, these are your patients, these are the people you're responsible for and it looks like the mortality rates higher than it should be, it's not just some fictional graph that got sent to you from central office, so it makes it a little bit more real” (TM11). One participant said that access to provider specific information would be helpful, since it’s well-known that comparing physicians to peers motivates them to improve their performance (TM09). If reports indicate a mortality rate at or below the mean, there shouldn’t be any complacency. Hospitals should still look for opportunities for improvement, although there is less urgency in doing so. In any case, the mortality rate could change quickly, because even just a few “disastrous things” could swing them in the other direction (TM08, TM10, TM11).

*Other uses*

Finally, other uses were mentioned by participants. Some would check documentation and coding (TM01) or might recommend that labs related to higher mortality be part of the panel of labs ordered for patients admitted to hospital or ICU (TM08). One participant said they needed to feel more comfortable with TM before knowing how to use it (TM04). Others said they need to understand more about what goes into the metric to decide where (and how) to spend energy improving things (TM05), and about the strengths and weaknesses of the underlying data before making decisions about programs, direction of programs, and/or resources to support programs, and so sometimes looks at data from two different sources to double check. If they are comfortable with data, they don’t have to look at multiple data sources (TM08). Finally, two participants offer criticisms: that they get a lot of reports, and so may not read all of them (TM03), or that they put more emphasis on internal benchmarking than external benchmarking (TM04).

**Appendix 5:** Summary of interview responses regarding opportunities for improvement

Regarding HS-TM, participants suggested alternate groupings to the 500 comparator hospitals. These included: disease categories, surgical vs non-surgical care, ICU deaths, and admissions sources such as transfers in, ED admissions, or clinic admissions. One participant pointed out that people may want to see different groupings depending on their role. For example, a center director might want to compare sites in their VISN to other VISNs, but a chief of medicine or ICU director might want to compare their ICU mortality to other ICUs. Participants also wanted more specificity with the comparator hospitals, such as comparing them based on region or type of hospital, such as comparing 1A to 1A or tertiary care to tertiary care. They also wanted race to be taken into consideration.

Participants generally agreed that mortality is not equivalent to quality of care, and that there are issues with how it is calculated and whether facilities can be compared based on it. Other quality measures they mentioned include:

- transfers to ICU
- length of stay
- readmission rates
- surgical complications
- patient satisfaction
- rapid response teams
- length of time people are intubated
- infection rates in different areas, e.g., surgery, ICUs, ORs, or on the floors
- type of infection, e.g., urinary, respiratory, or skin
- 7- and 30-day readmission rates
- out of hospital mortality rates
- complication rates

Participants requested additional information on the models, and changes to the performance reports. They wanted to know which patients are not included due to selection criteria, and where the data is coming from and how that data is acquired. One participant wanted an explanation of why the TM model is comparing to like facilities rather than all facilities, and another wanted the location of comparator hospitals to be included in the performance report. Another wanted CSI and case mix adjustment to be included on the graphs. A participant wanted the reports to indicate how many deaths would swing the hospital closer or further away from the mean.

Finally, there were several general recommendations. Others stated it would be helpful to compare VA hospitals to community hospitals. Some stated a preference for criterion reference over norm reference. Several mentioned that having provider-specific information would make the reports even more useful in enhancing motivation.

| **eTable 4**: Qualitative statements comparing the utility of HS-TM vs regression-based performance assessments, providing in response to the probe | |
| --- | --- |
| 1. | “clearly the [HS-TM] model addresses some of the concerns or refrain that we commonly hear from people that ‘oh I’m different and it's okay for me to ignore this measure because I’m different” |
| 2. | “I could feel confident that [HS-TM] is enhancing our ability to make an apples-to-apples comparison” |
| 3. | “I’m not sure how I would use [them] differently, but I might feel better about the data like it might have more meaning to me in [HS-TM].” |
| 4. | “I think that anytime I’m told that I’m a low performer in a model, you know I always go back and [ask] are there things I would quibble with? Yes, but these are minor enough issues that can't explain outlier status and I’m still going to do my own homework locally to better understand why we're a low performer in the model. And again, it would be surprising to me if that's being entirely driven by the fact that that we take care of such a unique patient population, or we provide such a complex service that we're getting referred the worst of the worst… it probably explains some of it.” |
| 5. | “maybe I’m too pragmatic I’m not sure that I would necessarily use it that differently” |
| 6. | “when you pull out information from the whole and then when you pull out information that might be from comparator hospitals that is a bit artificial. And so, the first method, I like seeing all the hospitals on there and seeing where I fit with all the hospitals” |
| These comments were provided in response to the probe, “would you use performance reports differently if they were in the form of template matching as compared to the current ones you get using regression?”. Overall, several interview participants expressed feeling more confident in the validity of HS-TM assessments, but participants nonetheless felt that regardless of the method they would use the data primarily as a screen for doing a deeper diver. | |

REFERENCES

1. Malterud K, Siersma VD, Guassora AD. Sample Size in Qualitative Interview Studies: Guided by Information Power. *Qual Health Res*. Nov 2016;26(13):1753-1760. doi:10.1177/1049732315617444

2. Marshall MN. Sampling for qualitative research. *Fam Pract*. Dec 1996;13(6):522-5. doi:10.1093/fampra/13.6.522

3. Hamilton AB, Finley EP. Qualitative methods in implementation research: An introduction. *Psychiatry Res*. Oct 2019;280:112516. doi:10.1016/j.psychres.2019.112516

4. Austin JM, McGlynn EA, Pronovost PJ. Fostering Transparency in Outcomes, Quality, Safety, and Costs. *JAMA*. 2016;316:1661-1662. doi:10.1001/jama.2016.14039

5. Pronovost PJ, Austin JM, Cassel CK, et al. Fostering Transparency in Outcomes, Quality, Safety, and Costs: A Vital Direction for Health and Health Care | National Academy of Medicine. 2016;

6. Lezzoni LI. The Risks of Risk Adjustment. *JAMA*. 1997;278:1600-1607. doi:10.1001/jama.1997.03550190064046

7. Silber JH, Rosenbaum PR, Ross RN, et al. Template matching for auditing hospital cost and quality. *Health Services Research*. 2014;49:1446-1474. doi:10.1111/1475-6773.12156

8. Hsieh HF, Shannon SE. Three approaches to qualitative content analysis. *Qual Health Res*. Nov 2005;15(9):1277-88. doi:10.1177/1049732305276687
